# Pharmacogenetic meta-analysis of genome-wide association studies of LDL cholesterol response to statins

**DOI:** 10.1038/ncomms6068

**Published:** 2014-10-28

**Authors:** Iris Postmus, Stella Trompet, Harshal A. Deshmukh, Michael R. Barnes, Xiaohui Li, Helen R. Warren, Daniel I. Chasman, Kaixin Zhou, Benoit J. Arsenault, Louise A. Donnelly, Kerri L. Wiggins, Christy L. Avery, Paula Griffin, QiPing Feng, Kent D. Taylor, Guo Li, Daniel S. Evans, Albert V. Smith, Catherine E. de Keyser, Andrew D. Johnson, Anton J. M. de Craen, David J. Stott, Brendan M. Buckley, Ian Ford, Rudi G. J. Westendorp, P. Eline Slagboom, Naveed Sattar, Patricia B. Munroe, Peter Sever, Neil Poulter, Alice Stanton, Denis C. Shields, Eoin O’Brien, Sue Shaw-Hawkins, Y.-D. Ida Chen, Deborah A. Nickerson, Joshua D. Smith, Marie Pierre Dubé, S. Matthijs Boekholdt, G. Kees Hovingh, John J. P. Kastelein, Paul M. McKeigue, John Betteridge, Andrew Neil, Paul N. Durrington, Alex Doney, Fiona Carr, Andrew Morris, Mark I. McCarthy, Leif Groop, Emma Ahlqvist, Welcome Trust Case Control Consortium, Joshua C. Bis, Kenneth Rice, Nicholas L. Smith, Thomas Lumley, Eric A. Whitsel, Til Stürmer, Eric Boerwinkle, Julius S. Ngwa, Christopher J. O’Donnell, Ramachandran S. Vasan, Wei-Qi Wei, Russell A. Wilke, Ching-Ti Liu, Fangui Sun, Xiuqing Guo, Susan R Heckbert, Wendy Post, Nona Sotoodehnia, Alice M. Arnold, Jeanette M. Stafford, Jingzhong Ding, David M. Herrington, Stephen B. Kritchevsky, Gudny Eiriksdottir, Leonore J. Launer, Tamara B. Harris, Audrey Y. Chu, Franco Giulianini, Jean G. MacFadyen, Bryan J. Barratt, Fredrik Nyberg, Bruno H. Stricker, André G. Uitterlinden, Albert Hofman, Fernando Rivadeneira, Valur Emilsson, Oscar H. Franco, Paul M. Ridker, Vilmundur Gudnason, Yongmei Liu, Joshua C. Denny, Christie M. Ballantyne, Jerome I. Rotter, L. Adrienne Cupples, Bruce M. Psaty, Colin N. A. Palmer, Jean-Claude Tardif, Helen M. Colhoun, Graham Hitman, Ronald M. Krauss, J Wouter Jukema, Mark J. Caulfield, Membership of Wellcome Trust Case Control Consortium, Peter Donnelly, Ines Barroso, Jenefer M. Blackwell, Elvira Bramon, Matthew A. Brown, Juan P. Casas, Aiden Corvin, Panos Deloukas, Audrey Duncanson, Janusz Jankowski, Hugh S. Markus, Christopher G. Mathew, Colin N. A. Palmer, Robert Plomin, Anna Rautanen, Stephen J. Sawcer, Richard C. Trembath, Ananth C. Viswanathan, Nicholas W. Wood, Data and Analysis Group, Chris C. A. Spencer, Gavin Band, Céline Bellenguez, Colin Freeman, Garrett Hellenthal, Eleni Giannoulatou, Matti Pirinen, Richard Pearson, Amy Strange, Zhan Su, Damjan Vukcevic, Peter Donnelly, DNA, Genotyping, Data QC and Informatics Group, Cordelia Langford, Sarah E. Hunt, Sarah Edkins, Rhian Gwilliam, Hannah Blackburn, Suzannah J. Bumpstead, Serge Dronov, Matthew Gillman, Emma Gray, Naomi Hammond, Alagurevathi Jayakumar, Owen T. McCann, Jennifer Liddle, Simon C. Potter, Radhi Ravindrarajah, Michelle Ricketts, Matthew Waller, Paul Weston, Sara Widaa, Pamela Whittaker, Ines Barroso, Panos Deloukas, Publications Committee, Christopher G. Mathew, Jenefer M. Blackwell, Matthew A. Brown, Aiden Corvin, Mark I. McCarthy, Chris C. A. Spencer

**Affiliations:** 1 Department of Gerontology and Geriatrics, Leiden University Medical Center, Leiden 2300 RC, The Netherlands; 2 The Netherlands Consortium for Healthy Ageing, Leiden 2300 RC, The Netherlands; 3 Department of Cardiology, Leiden University Medical Center, Leiden 2300 RC, The Netherlands; 4 Medical Research Institute, Ninewells Hospital and Medical School, University of Dundee, Dundee DD1 9SY, UK; 5 Genome Centre, William Harvey Research Institute, Barts and The London School of Medicine, Queen Mary University of London, London EC1M6BQ, UK; 6 NIHR Barts Cardiovascular Biomedical Research Unit, Queen Mary University of London, London EC1M 6BQ, UK; 7 Institute for Translational Genomics and Population Sciences, Los Angeles BioMedical Research Institute at Harbor-UCLA Medical Center, Torrance, California 90502, USA; 8 Department of Clinical Pharmacology, William Harvey Research Institute, Barts and The London School of Medicine, Queen Mary University of London, London EC1M6BQ, UK; 9 Division of Preventive Medicine, Brigham and Women's Hospital, Boston, Massachusetts 02215-1204, USA; 10 Harvard Medical School, Boston, Massachusetts 02215, USA; 11 Montreal Heart Institute, Universite de Montreal, Montreal H1T 1C8, Quebec, Canada; 12 Cardiovascular Health Research Unit, Department of Medicine, University of Washington, 98101 Seattle, Washington, USA; 13 Department of Epidemiology, University of North Carolina, Chapel Hill, North Carolina 27599, USA; 14 Department of Biostatistics, Boston University School of Public Health, Boston, Massachusetts 02215, USA; 15 Department of Clinical Pharmacology, Vanderbilt University, Nashville, Tennessee 37240, USA; 16 California Pacific Medical Center Research Institute, San Francisco, California 94107, USA; 17 Icelandic Heart Association, IS-201 Kopavogur, Iceland; 18 University of Iceland, IS-101 Reykjavik, Iceland; 19 Department of Epidemiology, Erasmus Medical Center, 3000 CA Rotterdam, The Netherlands; 20 Health Care Inspectorate, 2595 AN The Hague, The Netherlands; 21 Framingham Heart Study (FHS) of the National Heart, Lung and Blood Institute, Cardiovascular Epidemiology and Human Genomics, Framingham, Massachusetts 01702, USA; 22 Faculty of Medicine, Institute of Cardiovascular and Medical Sciences, University of Glasgow, Glasgow G31 2ER, UK; 23 Department of Pharmacology and Therapeutics, University College Cork, Cork 30, Ireland; 24 Robertson Center for Biostatistics, University of Glasgow, Glasgow G12 8QQ, UK; 25 Leyden Academy of Vitality and Ageing, 2333 AA Leiden, The Netherlands; 26 Department of Molecular Epidemiology, Leiden University Medical Center, 2300 RC Leiden, The Netherlands; 27 Faculty of Medicine, BHF Glasgow Cardiovascular Research Centre, Glasgow G12 8QQ, UK; 28 International Centre for Circulatory Health, Imperial College, London SW7 2AZ, UK; 29 Molecular and Cellular Therapeutics, Royal College of Surgeons in Ireland, Dublin 2, Ireland; 30 Beaumont Hospital, Dublin 9, Ireland; 31 The Conway Institute, University College Dublin, Dublin 4, Ireland; 32 School of Medicine and Medical Sciences, University College Dublin, Dublin 4, Ireland; 33 Department of Genome Sciences, University of Washington, Seattle, Washington 98101, USA; 34 Department of Cardiology, Academic Medical Center, 1100 DD Amsterdam, The Netherlands; 35 Department of Vascular Medicine, Academic Medical Center, 1100 DD Amsterdam, The Netherlands; 36 University of Edinburgh, Edinburgh EH9 3JR, UK; 37 University College, London WC1E 6BT, UK; 38 University of Oxford, Oxford OX1 2JD, UK; 39 Cardiovascular Research Group, School of Biosciences, University of Manchester, Manchester M13 9NT, UK; 40 Oxford Centre for Diabetes, Endocrinology and Metabolism, University of Oxford, Churchill Hospital, Old Road, Headington, Oxford OX3 7LJ, UK; 41 Wellcome Trust Centre for Human Genetics, University of Oxford, Roosevelt Drive, Oxford OX3 7BN, UK; 42 Oxford NIHR Biomedical Research Centre, Churchill Hospital, Old Road, Headington, Oxford OX3 7LJ, UK; 43 Department of Clinical Sciences/Diabetes & Endocrinology, Lund University, Malmo 205 02, Sweden; 44 Department of Biostatistics, University of Washington, 98115 Seattle, Washington, USA; 45 Department of Epidemiology, University of Washington, Seattle, Washington 98195, USA; 46 Group Health Research Institute, Group Health Cooperative, Seattle, Washington 98101, USA; 47 Seattle Epidemiologic Research and Information Center, Department of Veterans Affairs Office of Research and Development, Seattle, Washington 98101, USA; 48 Department of Statistic, University of Auckland, Auckland 1142, New Zealand; 49 Department of Medicine, University of North Carolina, Chapel Hill, North Carolina 27599, USA; 50 Human Genetics Center, School of Public Health, University of Texas Health Science Center at Houston, Houston, Texas 77030, USA; 51 NHLBI Framingham Heart Study, Framingham, Massachusetts 01701, USA; 52 Cardiology Division, Department of Medicine, Massachusetts General Hospital, Harvard Medical School, Boston, Massachusetts 02115, USA; 53 National Heart, Lung and Blood Institute, Bethesda, Maryland 20892, USA; 54 Section of Preventive Medicine and Epidemiology, Department of Medicine, Boston University School of Medicine, and the Framingham Heart Study, Framingham, Massachusetts 01701, USA; 55 Department of Biomedical Informatics, Vanderbilt University, Nashville, Tennessee 37240, USA; 56 Department of Internal Medicine, Center for IMAGENETICS, Sanford Healthcare, Fargo, North Dakota, 58104 USA; 57 Cardiovascular Health Research Unit, University of Washington, Seattle, Washington 98101, USA; 58 Department of Cardiology, Johns Hopkins University, Baltimore, Maryland 21218, USA; 59 Division of Cardiology, Harborview Medical Center, University of Washington, Seattle 98101, Washington, USA; 60 Division of Public Health Sciences, Department of Biostatistical Sciences, Wake Forest School of Medicine, Winston-Salem, North Carolina 27157, USA; 61 Division of Public Health Sciences, Department of Epidemiology and Prevention, Wake Forest School of Medicine, Winston-Salem, North Carolina 27157, USA; 62 Department of Internal Medicine, Section on Cardiology, Wake Forest School of Medicine, Winston-Salem, North Carolina 27157, USA; 63 Department of Internal Medicine, Wake Forest School of Medicine, Winston-Salem, North Carolina 27157, USA; 64 Laboratory of Epidemiology, Demography, Biometry, National Institute on Aging, National Institutes of Health, 7201 Wisconsin Avenue, Bethesda, Maryland 20892, USA; 65 Personalised Healthcare and Biomarkers, AstraZeneca, Alderley Park SK10 4TG, UK; 66 AstraZeneca Research and Development, 481 83 Mölndal, Sweden; 67 Unit of Occupational and Environmental Medicine, University of Gothenburg, 405 30 Gothenburg, Sweden; 68 Department of Internal Medicine, Erasmus Medical Center, 3000 CA Rotterdam, The Netherlands; 69 Department of Medicine, Vanderbilt University, Vanderbilt, Tennessee 37240, USA; 70 Department of Medicine, Baylor College of Medicine, Houston, Texas 77030, USA; 71 Department of Health Services, University of Washington, Seattle, Washington 98101, USA; 72 Department of Public Health, University of Dundee, Dundee DD1 9SY, UK; 73 Barts and the London School of Medicine and Dentistry, Queen Mary University of London, London E1 2AT, UK; 74 Children’s Hospital Oakland Research Institute, Oakland, California 94609, USA; 75 Durrer Center for Cardiogenetic Research, 1105 AZ Amsterdam, The Netherlands; 76 Interuniversity Cardiology Institute of the Netherlands, 3511 GC Utrecht, The Netherlands; 77 Wellcome Trust Centre for Human Genetics, Roosevelt Drive, Oxford, UK; 78 Department of Statistics, University of Oxford, Oxford, UK; 79 Wellcome Trust Sanger Institute, Wellcome Trust Genome Campus, Hinxton, Cambridge, UK; 80 Telethon Institute for Child Health Research, Centre for Child Health Research, University of Western Australia, 100 Roberts Road, Subiaco, Western Australia, Australia; 81 Cambridge Institute for Medical Research, University of Cambridge School of Clinical Medicine, Cambridge, UK; 82 Department of Psychosis Studies, NIHR Biomedical Research Centre for Mental Health at the Institute of Psychiatry, King’s College London and The South London and Maudsley NHS Foundation Trust, Denmark Hill, London, UK; 83 University of Queensland Diamantina Institute, Princess Alexandra Hospital, University of Queensland, Brisbane, Queensland, Australia; 84 Department of Epidemiology and Population Health, London School of Hygiene and Tropical Medicine, London, UK; 85 Department of Epidemiology and Public Health, University College London, London, UK; 86 Neuropsychiatric Genetics Research Group, Institute of Molecular Medicine, Trinity College Dublin, Dublin, Ireland; 87 Molecular and Physiological Sciences, The Wellcome Trust, London, UK; 88 Centre for Digestive Diseases, Queen Mary University of London, London, UK; 89 Digestive Diseases Centre, Leicester Royal Infirmary, Leicester, UK; 90 Department of Clinical Pharmacology, Old Road Campus, University of Oxford, Oxford, UK; 91 Clinical Neurosciences, St George’s University of London, London, UK; 92 King’s College London, Department of Medical and Molecular Genetics, School of Medicine, Guy’s Hospital, London, UK; 93 Medical Research Institute, University of Dundee, Ninewells Hospital and Medical School, Dundee, UK; 94 King’s College London Social, Genetic and Developmental Psychiatry Centre, Institute of Psychiatry, Denmark Hill, London, UK; 95 University of Cambridge, Department of Clinical Neurosciences, Addenbrooke’s Hospital, Cambridge, UK; 96 NIHR Biomedical Research Centre for Ophthalmology, Moorfields Eye Hospital NHS Foundation Trust and UCL Institute of Ophthalmology, London, UK; 97 Department of Molecular Neuroscience, Institute of Neurology, Queen Square, London, UK; 98 Oxford Centre for Diabetes, Endocrinology and Metabolism (ICDEM), Churchill Hospital, Oxford, UK

## Abstract

Statins effectively lower LDL cholesterol levels in large studies and the observed interindividual response variability may be partially explained by genetic variation. Here we perform a pharmacogenetic meta-analysis of genome-wide association studies (GWAS) in studies addressing the LDL cholesterol response to statins, including up to 18,596 statin-treated subjects. We validate the most promising signals in a further 22,318 statin recipients and identify two loci, *SORT1/CELSR2/PSRC1* and *SLCO1B1*, not previously identified in GWAS. Moreover, we confirm the previously described associations with *APOE* and *LPA.* Our findings advance the understanding of the pharmacogenetic architecture of statin response.

## Abstract (web summary)

Statins are effectively used to prevent and manage cardiovascular disease, but patient response to these drugs is highly variable. Here, the authors identify two new genes associated with the response of LDL cholesterol to statins and advance our understanding of the genetic basis of drug response.

## Graphical abstract

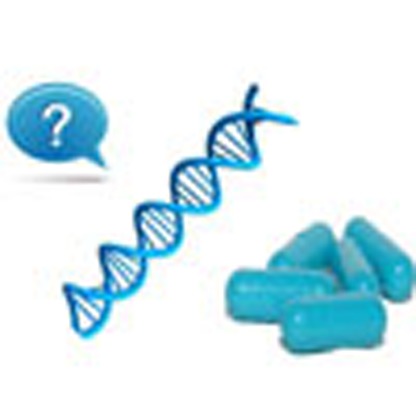

The 3-hydroxymethyl-3-methylglutaryl coenzyme A (HMG-CoA) reductase inhibitors, also known as statins, are widely prescribed and are highly effective in the management and prevention of cardiovascular disease. Statin therapy results in a lowering of low-density lipoprotein cholesterol (LDL-C) levels by up to 55%^1^ and a 20–30% reduction of cardiovascular events^2^. Despite the clinical efficacy of statins in a wide range of patients^2^, interindividual variability exists with regard to LDL-C-lowering response as well as efficacy in reducing major cardiovascular events^3^. The suggestion that some of this variability may be due, in part, to common pharmacogenetic variation is supported by previous studies that have identified genetic variants associated with differential LDL-C response to statin therapy^4^,^5^,^6^.

A small number of genome-wide association studies (GWAS) have previously identified loci associated with statin response on a genome-wide level. A GWAS in the JUPITER trial identified three genetic loci, *ABCG2* (rs2199936), *LPA* (rs10455872) and *APOE* (rs7412), that were associated with percentage LDL-C reduction following rosuvastatin therapy^7^. In the CARDS and ASCOT studies, single nucleotide polymorphisms (SNPs) at *LPA* (rs10455872) and *APOE* (rs445925 and rs4420638) were associated with LDL-C response to atorvastatin treatment^8^. A combined GWAS in three statin trials identified a SNP within *CLMN* (rs8014194) that is associated with the magnitude of statin-induced reduction in plasma cholesterol^9^. However, two other GWAS identified no genetic determinants of LDL-C response to statin therapy at a genome-wide significant level^6^,^10^.

On the basis of these studies, as well as previous candidate gene studies^4^,^6^, the only genetic variants that have been consistently identified to be associated with variation in LDL-C response to statin therapy, irrespective of statin formulation, are located at or nearby *APOE* and *LPA*. To determine whether additional loci may influence LDL-C response to statins, we formed the Genomic Investigation of Statin Therapy (GIST) consortium and conducted a pharmacogenetic meta-analysis using GWAS data sets from randomized controlled trials (RCTs) and observational studies. We identify two loci not previously identified in GWAS, *SORT1/CELSR2/*PSRC1 and *SLCO1B1.* In addition, we confirm the associations within the *APOE* and *LPA* genes. These findings will extend the knowledge of the pharmacogenetic architecture of statin response.

## Results

### First-stage meta-analysis

The GIST consortium includes 6 RCTs (*n*=8,421 statin recipients) and 10 observational studies (*n*=10,175 statin recipients) that participated in the first stage (see Methods; Supplementary Tables 1 and 2; Supplementary Notes 1 and 2). To search for genetic variants associated with differential LDL-C response to statin therapy, each study independently performed a GWAS among statin users, using the difference between the natural log-transformed LDL-C levels on- and off-treatment as the response variable (see Methods).

**Figure 1:**
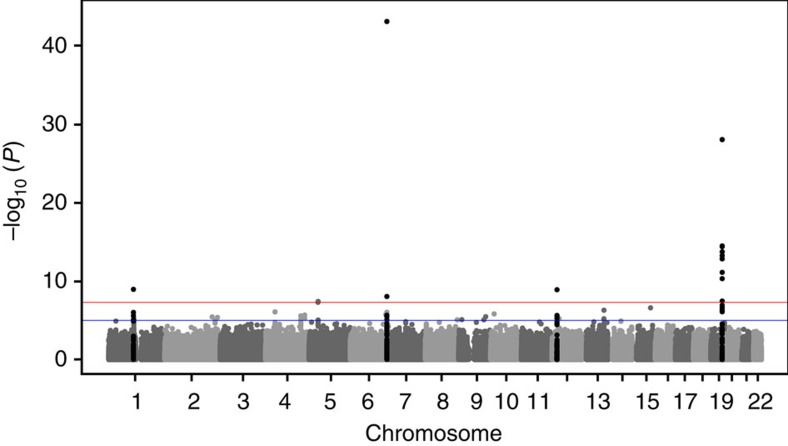
Results of the GWAS meta-analysis. Manhattan plot presenting the −log_10_ *P* values from the combined meta-analysis (*n*=40,914) on LDL-C response after statin treatment. *P* values were generated using linear regression analysis.

**Figure 2:**
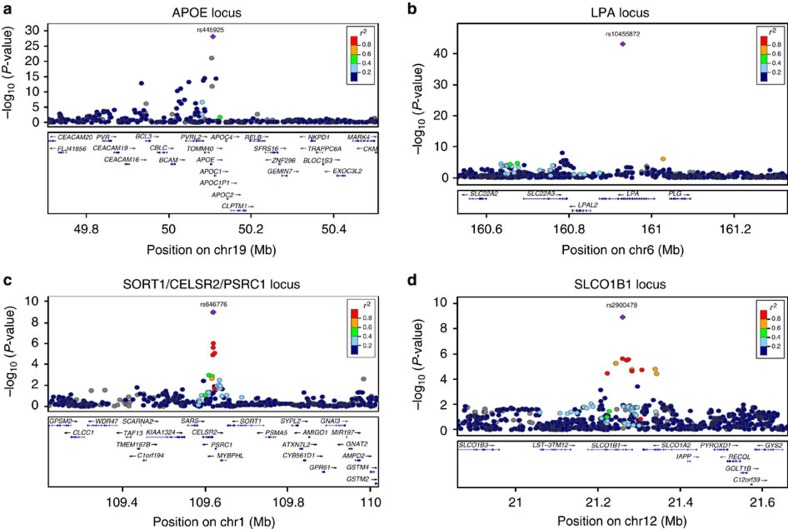
Regional association plots of the genome-wide significant associations with LDL-C response after statin treatment. The plots show the genome-wide significant associated loci in the combined meta-analysis (*n*=40,914), the *APOE* locus (**a**), the *LPA* locus (**b**), the *SORT1/CELSR2/PSRC1* locus (**c**) and the *SLCO1B1* locus (**d**) (generated using LocusZoom ( http://genome.sph.umich.edu/wiki/LocusZoom)). The colour of the SNPs is based on the LD with the lead SNP (shown in purple). The RefSeq genes in the region are shown in the lower panel. *P* values were generated using linear regression analysis.

The first-stage meta-analysis identified three loci, including 13 SNPs, that attained genome-wide significance (*P*<5 × 10^−8^) for association with LDL-C response to statin treatment (Fig. 1; Table 1). The most significant association was for a SNP on chromosome 19, at *APOE* (rs445925, minor allele frequency (MAF)=0.098, *β*=−0.043, s.e.=0.005, *P*=1.58 × 10^−18^; Fig. 2a), indicating that carriers of the rs445925 SNP respond to statins with an additional 4.3% increase per allele in LDL-C lowering effect compared with non-carriers. The second strongest association was with a SNP at *LPA* on chromosome 6 (rs10455872, MAF=0.069, *β*=0.041, s.e.=0.006, *P*=1.95 × 10^−11^; Fig. 2b), indicating a 5.9% smaller LDL-C lowering per minor allele for carriers of the SNP compared with non-carriers. Associations at both loci have previously been described^7^,^8^. A third genome-wide significant association was found with a SNP at *RICTOR* on chromosome 5 (rs13166647, MAF=0.230, *β*=−0.253, s.e.=0.046, *P*=4.50 × 10^−8^), although genotypes for this SNP were only available in two studies within the first stage (*n*=2,144).

### Second-stage meta-analysis

We selected 246 SNPs with *P* <5 × 10^−4^ from 158 loci for further investigation in three additional studies comprising up to 22,318 statin-treated subjects (see Methods; Supplementary Tables 1 and 5; Supplementary Note 3). This second stage confirmed the genome-wide significant associations between variations within the *APOE* and *LPA* loci and LDL-C response, as observed in the first stage (Table 1; Supplementary Fig. 2; Supplementary Table 5). In addition, SNPs at two new loci with *P* values between 6.70 × 10^−7^ and 2.26 × 10^−6^ in the first phase were shown to be significantly associated with statin-induced LDL-C lowering after statin treatment in the total combined meta-analysis at a genome-wide level: *SORT1/CELSR2/PSRC1* (rs646776, *β*=−0.013, s.e.=0.002, *P*=1.05 × 10^−9^ and rs12740374, *β*=−0.013, s.e.=0.002, *P*=1.05 × 10^−9^; Fig 2c) and *SLCO1B1* (rs2900478, *β*=0.016, s.e.=0.003, *P*=1.22 × 10^−9^; Fig 2d), indicating an additional 1.5% increase per allele in LDL-C lowering effect for carriers of the *SORT1/CELSR2/PSRC1* SNP and a 1.6% smaller LDL-C lowering per minor allele for carriers of the *SLCO1B1* SNP.

The six next-ranked SNPs with *P* values just below 5 × 10^−8^ in the combined meta-analysis, including the two SNPs at *RICTOR* (rs13166647 and rs13172966), were selected for additional genotyping in the Scandinavian ASCOT participants (see Methods). None of these six SNPs reached genome-wide significance after this additional genotyping (Supplementary Table 6). Therefore, our overall genome-wide significant findings were the SNPs at *APOE*, *LPA*, *SORT1/CELSR2/PSRC1* and *SLCO1B1*.

### Subfraction analyses

To extend our results for the novel GWAS finding *SORT1/CELSR2/PSRC1*, we performed additional association analyses, using measurements of cholesterol levels in four LDL subfractions (large, medium, small and very small) from two of the trials in GIST, CAP and PRINCE (Table 2; see Methods). The minor allele of *SORT1* rs646776 was associated with greater statin-induced reductions in levels of all LDL subfractions, and there was a nonsignificant trend for larger effect sizes and greater statistical significance for lowering of small and very small LDL (Table 2). In contrast, the *APOE* SNP associated with greater LDL-C response to statins (rs445925) showed a small and nonsignificant association with change in very small LDL (Table 2). For the minor allele of rs2900478 (*SLCO1B1*), the borderline significant association with smaller magnitude of LDL-C reduction showed a trend for preferential association with larger versus smaller LDL subfractions. The lack of association of rs10455872 (*LPA*) with changes in LDL subfractions is consistent with evidence discussed below that this locus affects levels of lipoprotein(a) (Lp(a)) and not LDL particles. Using generalized estimating equations, we tested the association of log change in each of the LDL subfractions with interactions of the four SNPs. For very small LDL, the association with the rs646776 minor allele was significantly different from that of the other minor alleles (*P*=0.03 after adjustment for multiple testing).

### Effects of off-treatment LDL-C

To demonstrate that our findings for LDL-C response to statin treatment are unlikely to be explained through associations with baseline LDL-C levels, we performed a number of additional analyses (see Methods). First, Supplementary Table 7 shows regression coefficients for baseline-adjusted and measurement noise-corrected estimates of the direct effect of genotype on on-treatment LDL-C at the strongest SNPs in the GIST meta-analysis (*P*<1 × 10^−8^), which were available in the CARDS data set. Correcting our effect size estimate further and modelling measurement noise at baseline reduced the apparent effect only slightly for all the markers, suggesting that there is little effect of measurement noise. Next, within the JUPITER trial, additional analyses were performed to determine whether there was an interaction between LDL-C change and statin or placebo allocation. Supplementary Table 8 shows significant *P* values for interaction (all <5 × 10^−2^) for SNPs at the four genome-wide significant loci in the GIST meta-analysis, also suggesting that genetic effects on baseline LDL-C as manifested in the placebo group contribute at most only in part to genetic effects on LDL-C response in the statin group.

### Genome-Wide Conditional Analysis

To investigate whether there were multiple SNPs within any gene and multiple loci associated with differential LDL-C lowering to statin therapy, we performed a conditional analysis across the genome using the summary statistics of the combined meta-analysis. The results of the Genome-Wide Conditional Analysis (GWCA; see Methods; Supplementary Table 9) showed 14 SNPs independently associated with statin response and these explained ~5% of the variation in LDL-C response to statin treatment. Of the 14 independent SNPs, 6 were genome-wide significant in the combined GWAS meta-analysis (Supplementary Table 5).

### Previous findings

In Supplementary Table 10, we performed a look-up in our GWAS meta-analysis for SNPs previously described in the literature (NHGRI Catalogue^11^ of Published GWAS and Candidate gene studies) to be associated with statin response, besides the loci associated at a genome-wide level in the current study. None of these SNPs was associated with statin response in our GWAS after correcting for multiple testing.

### Functional analyses

Functional characterization of the 246 SNPs selected for the second stage was performed using a range of bioinformatics tools (see Methods). A total of 420 expression quantitative trait loci (eQTL) associations were identified across a wide range of tissues (Supplementary Data 1), which comprised 67 independent gene eQTL associations. Eleven genes, including *APOE*, *SORT1*, *CELSR2* and *PSRC1*, showed eQTLs in liver, which considering its primary role in mediating statin-induced LDL reduction may be particularly relevant to statin response. Putative gene eQTLs were combined with genes annotated to variants in linkage disequilibrium (LD) with LDL-C response-associated variants, resulting in a list of 185 candidate gene loci, defined by 2,681 SNPs (Supplementary Data 2 and 3). To identify statin responsive genes among the candidate loci, gene expression data measured in response to statin treatment in a range of cell lines was retrieved from the Connectivity Map resource^12^ (see Methods). Five genes (*APOE, BRCA1, GRPEL1, ADRB2* and *ETV1*) showed convincing evidence of statin responsiveness on the basis of greater than twofold differential expression in response to statin treatment. Eight genes showed suggestive evidence (1.5- to 2-fold change; *TOMM40*, *SREBP1*, *PSRC1*, *BCL3*, *BCAM*, *ANK3*, *SIVA1* and *RANBP9*; Supplementary Data 3).

Finally, involvement in statin response was investigated at a pathway level using GeneGo Metacore (Thomson Reuters^13^). Briefly, 87 literature-reported genes linked to statin response were combined with the 185 candidate gene loci reported here (Supplementary Data 3). A conservative network of direct interactions was constructed between query genes (Supplementary Data 4). The network included 24 genes located in the LDL-C-associated loci (Supplementary Fig. 4). Collectively, our functional and pathway analysis confirms a strong biological and functional role in statin response for several strongly associated gene loci, including *APOE/TOMM40/PVRL2* and *SORT1/CELSR2/PSRC2.*

## Discussion

We have performed a meta-analysis of GWAS including more than 40,000 subjects, investigating genetic variants associated with variation in LDL-C lowering on statin treatment independent from associations with baseline LDL-C. We identified four loci at genome-wide significance, including the previously identified *APOE* and *LPA,* and the novel GWAS loci *SORT1/CELSR2/PSRC1* and *SLCO1B1*.

Nine SNPs in the *APOE* gene region reached genome-wide significance for LDL-C response. The minor allele of the lead SNP rs445925, which is a proxy for the apoE ε2 protein variant defining SNP rs7412 (ref. 14)^14^, was associated with a larger LDL-C-lowering response to statins compared with carriers of the major allele. The magnitude and direction of the effect size was similar to previously reported findings for the rs445925 variant in the GWAS study performed in CARDS and ASCOT^8^ and of the SNP rs7412 in JUPITER^7^. Since the apoE ε2 protein results in increased hepatic cholesterol synthesis, it may also predispose to stronger inhibition of cholesterol synthesis by statin treatment^8^,^10^.

Three independent SNPs at *LPA* were significantly associated with LDL-C response to statins. The minor G allele of the lead SNP rs10455872 was associated with smaller LDL-C reduction than the major allele. This result was similar to the previous GWAS findings for this SNP in the JUPITER trial and the combined ASCOT and CARDS study^7^,^8^. The rs10455872 SNP was strongly associated with the KIV-2 copy number variant in Lp(a), which encodes variability in apo(a) size and is responsible for ~30% of variance in Lp(a) levels^8^,^15^. Furthermore, rs10455872 was shown to be strongly associated with plasma Lp(a) levels^16^. Standard assays of LDL-C, as well as the Friedewald formula, include cholesterol that resides in Lp(a)^6^,^8^. Carriers of this *LPA* variant are characterized by higher Lp(a) levels and a larger proportion of their measured LDL-C resides in Lp(a) particles^8^,^10^. Since statin therapy does not reduce the number of Lp(a) particles^17^, their presence attenuates the measured LDL-C response to statins.

Two SNPs at *SORT1/CELSR2/PSRC1* (rs646776 and rs12740374) on chromosome 1p were associated with an enhanced statin LDL-C response. A similar association was previously observed in a large candidate gene study in HPS^6^; however, we demonstrate this finding now first at a genome-wide significance level. The minor allele of rs12740374 has been shown to generate a binding site for the transcription factor C/EBPa^18^. Transcription results in upregulation of hepatic expression of three genes at this locus, *SORT1*, *CELSR2* and *PSRC1* (ref. 18), which we also showed in our eQTL analysis (Supplementary Data 1). Of these, *SORT1* is most notable, in that it encodes the multifunctional intracellular trafficking protein sortilin, which has been shown to bind tightly to apoB^19^. Sortilin-induced lowering of plasma LDL-C results from two mechanisms: reduced secretion of apoB-containing precursors, and, perhaps of greater importance, increased hepatic LDL uptake via binding to sortilin at the cell surface, with subsequent internalization and lysosomal degradation^19^. Notably, the minor allele of rs646776 is preferentially associated with lower levels of small and very small LDL (Table 2), suggesting that sortilin is of particular importance for regulating levels of these particles^18^. Smaller LDL subfractions have been shown to be relatively enriched in particles with reduced LDL receptor binding affinity and cellular uptake^20^, a property that may contribute to their associations with increased risk for cardiovascular disease^21^. This property may also underlie the diminished efficacy of statins for reduction of these particles (Supplementary Fig. 3)^22^, since statins act to reduce LDL-C levels to a large extent by increasing LDL receptor expression as a result of upregulation of the transcription factor SREBP2, whereas *SORT1* is not regulated by this mechanism. Hence, the greater statin-mediated reduction of LDL-C among carriers of the rs646776 minor allele could be attributed to relative depletion of LDL particles dependent on sortilin for clearance and hence a residually greater proportion of those LDL particles whose uptake is more dependent on the LDL receptor than on sortilin.

Notably, the strong association of rs646776 with statin-induced reductions in small and very small LDL particles contrasts to the weaker associations of changes in these particles with rs445925, likely the result of differing mechanisms underlying the effects of these SNPs on statin response. As noted above, rs445925 is a proxy for the SNP defining the apoE ε2 protein variant that is thought to predispose to heightened statin response as a result of greater statin inhibition of cholesterol synthesis and hence upregulation of SREBP and LDL receptor activity.

The *SLCO1B1* rs2900478 minor allele was associated with a smaller LDL-C reduction in response to statin treatment. *SLCO1B1* encodes the organic anion-transporting polypeptide OATP1B1 and facilitates the hepatic uptake of statins^23^. SNP rs2900478 is in strong LD (*r*^2^=0.89) with rs4149056, which represents the Val174Ala substitution resulting in complete loss of function. In the HPS trial, which used simvastatin, this candidate gene SNP was associated with a 1% lower LDL-C reduction per allele^6^. Single-dose studies have shown that the observed area under the curve of plasma level of active simvastatin after a dose of 40 mg was 221% higher in rs4149056 CC homozygotes compared with rs4149056 TT homozygotes, as compared with atorvastatin 20 mg (144% higher for CC versus TT) and rosuvastatin 40 mg (117% higher for CC versus TT)^24^. This finding results from the slower hepatic uptake of statins caused by the genetic variant, which would also be expected to result in a reduction in the cholesterol-lowering effect^25^. In a GWAS of the genetic risk factors for simvastatin-induced myopathy, *SLCO1B1* showed the strongest association^25^. Homozygous carriers of the *SLCO1B1* variant had a 16.9 times higher risk for myopathy compared with non-carriers. This might have led to a decrease in study medication adherence, and consequently a decreased effect on LDL-C in carriers of this SNP. In addition, previous analysis in the GoDARTS study showed that the effect of the *SLCO1B1* gene on statin efficacy was abolished after removal of individuals who showed signs of intolerance^26^.

GWCA identified three independent loci in the *APOE* gene region and two loci in the *LPA* gene region (Supplementary Table 9). GWCA also showed several other loci with *P* <5 × 10^−8^ that were not GWAS significant on single-SNP analysis (*HGD*, *RNF175*, *ISCA1L-HTR1A*, *GLIS3-SLC1A1*, *LOC100128657*, *NKX2-3-SLC25A28* and *PELI2*). These findings will require replication in independent, larger data sets. The significant SNPs in the GWCA analysis explained ~5% of the variation in LDL-C response to statin treatment. Whether this 5% is clinically relevant should be investigated by other studies. For example, it would be of interest to investigate whether this differential LDL-C lowering is also associated with differential event reduction by statin treatment.

In the current study, we combined the results of 6 randomized clinical trials and 10 observational studies in the first stage. This approach resulted also in combining several types of statins, since different statins were studied in the trials and within the observational studies (Supplementary Table 2). This, and the variation in statin dosage during follow-up for an individual, is a limitation of the current study, since, for example, the impact of the *SLCO1B1* variant on statin pharmacogenetics is known to be highly dependent on statin type and dose^24^,^27^. To overcome this limitation, the individual study analyses were adjusted for statin dose. Dividing the actual statin dose given by the statin-specific dose equivalent (Supplementary Table 3) gives the statin-adjusted equivalent based on the daily dosages required to achieve a mean 30% LDL-C reduction. Using this table, we made the different statin dosages and types comparable within the studies. To correct for between-study variance, we used a fixed effect meta-analysis with inverse variance weighting. Since we observed that the *SLCO1B1* gene was genome-wide significantly associated with LDL lowering, this highlights the thoroughness of our analytical approach, in which the analyses were correctly adjusted for the type and dose of statins used (Supplementary Table 3). Moreover, a comparison of the estimates of the SNPs between the RCTs (where there are no intra-individual differences in dosages) with the estimates of the SNPs in the observational studies showed large homogeneity between the estimates in the various study designs (Supplementary Fig. 2), indicating that our adjustment for dosage seems to be sufficient within this study.

Another possible limitation of the current study is the influence of the identified genetic variants on baseline LDL-C levels. In pharmacogenetic studies investigating the LDL-C-lowering response to statins, it is important to eliminate the effect of association between the genetic variant and baseline LDL-C levels, since those findings may confound the response to treatment associations. Previous large GWAS studies have shown strong associations between baseline LDL-C levels and genetic variants in *SORT1/CELSR2/PSRC1*, *APOE* and *LPA*^28^. To eliminate those possible confounding effects, our response to treatment analyses were adjusted for baseline LDL-C levels. In addition, additional analysis in CARDS and JUPITER suggests no or little influence of genetic associations with baseline LDL-C on the genetic effects on LDL-C-lowering response.

In conclusion, this study is the largest meta-analysis of GWAS for LDL-C response to statin therapy conducted to date. Our results demonstrate that apart from the previously identified *APOE* and *LPA* loci, two new loci, *SORT1/CELSR2/PSRC1* and *SLCO1B1*, also have a modest but genome-wide significant effect on LDL-C response. The minor alleles of the *APOE* rs445925 and *SORT1*/*CELSR2/PSRC1* rs646776 SNPs were associated with a larger statin response, whereas the minor alleles of the *LPA* rs10455872 and *SLCO1B1* rs2900478 SNPs were associated with a smaller statin response. Our findings advance the understanding of the pharmacogenetic architecture of statin response.

## Methods

### Study populations

The meta-analysis was conducted in the GIST consortium, which includes data from 8 randomized controlled statin trials (RCTs) and 11 prospective, population-based studies. The initial analysis (first stage) was performed in 8,421 statin-treated subjects from 6 RCTs (ASCOT, CARDS, CAP, PRINCE, PROSPER and TNT) and 10,175 statin-treated subjects from 10 observational studies (AGES, ARIC, BioVU, CHS, FHS, GoDARTS I, GoDARTS II, Health ABC, HVH and MESA). Further investigation (second stage) was performed in 21,975 statin-treated subjects from two randomized trials (HPS and JUPITER) and one observational study (Rotterdam Study). Six SNPs were additionally genotyped in the Scandinavian participants of the ASCOT study. The details of the first- and second-stage studies can be found in the Supplementary Tables 1 and 2 and Supplementary Notes 1 and 2.

### Subjects

Response to statin treatment was studied in statin-treated subjects only and not in those treated with placebo. Subjects included in the observational studies’ analysis should be treated with statins and have LDL-C measurements before and after start of statin treatment. Subjects of reported or suspected non-European ancestry were excluded. All participants gave written informed consent and the study was approved by all institutional ethics committees.

### Outcome measurements

The response to statin treatment was defined as the difference between the natural log-transformed on- and off-treatment LDL-C levels. The beta of the corresponding regression thus reflects the fraction of differential LDL lowering in carriers versus non-carriers of the SNP. For observational studies, the on-treatment LDL-C levels were taken into account for all kinds of prescribed statins, at any dosage, for any indication and for at least 4 weeks before measurement. Characteristics of on- and off-treatment LDL-C levels and statins used in each study are shown in Supplementary Table 2. For each individual, at least one off-treatment LDL-C measurement and at least one on-treatment LDL-C measurement were required. When multiple on- or off-treatment measurements were available, the mean of the cholesterol measurements was used. Subjects with missing on- or off-treatment measurements were excluded, with the exception of the GoDARTS cohorts for which missing off-treatment LDL-C levels were estimated using imputation methods (Supplementary Note 2). In the HPS, proportional LDL-C response was defined by the changes in natural log lipid levels from the screening visit before starting statin therapy to the randomization visit^6^.

### Genotyping and imputation

Genotyping, quality control, data cleaning and imputation were performed independently in each study using different genetic platforms and software as outlined in Supplementary Table 4. In all studies, genotyping was performed using Illumina, Affymetrix or Perlegen genotyping arrays, and MACH, Impute or BIMBAM software was used for imputation.

### GWAS analysis

Each study independently performed the GWAS on the difference between natural log-transformed on- and off-treatment LDL-C levels. To control for possible associations with off-treatment LDL-C levels, analyses were adjusted for the natural log-transformed off-treatment LDL-C level. An additive genetic model was assumed and tested using a linear regression model. For imputed SNPs, regression analysis was performed onto expected allele dosage. Analyses were additionally adjusted for age-, sex- and study-specific covariates (for example, ancestry principal components or country). Analyses in the observational studies were, if available, additionally adjusted for the statin dose by the natural logarithm of the dose equivalent as defined in Supplementary Table 3. This table shows the dose equivalent per statin type; dividing the statin dosage of an individual by the dose equivalent shown in Supplementary Table 3 will give the adjusted statin dosage.

### Quality control and meta-analysis

Centrally, within each study, SNPs with MAF <1% or imputation quality <0.3 were excluded from the analysis. QQ-plots were assessed for each study to identify between-study differences (Supplementary Fig. 1). The software package METAL was used for performing the meta-analysis ( http://www.sph.umich.edu/csg/abecasis/Metal/index.html). A fixed effects, inverse variance weighted approach was used. Using an inverse variance weighted meta-analysis will give smaller weights to studies with large s.e.. To correct for possible population stratification, genomic control was performed by adjusting the within-study findings and the meta-analysis results for the genomic inflation factor.

### Second stage

SNPs with *P* values <5 × 10^−4^ in the first-stage meta-analysis were selected for further investigation in a second stage. A maximum of two SNPs per locus were selected, based on statistical significance, except for the *APOE* locus, for which all genome-wide significant associated SNPs were selected for validation. A total of 246 SNPs, within 158 independent loci, were selected for the second stage, which was performed in the JUPITER trial, HPS study and the Rotterdam Study, which all had GWAS data and response to statin treatment available. For 2 of the 246 SNPs, a proxy was used in the JUPITER trial, and 31 SNPs were not available, nor was a proxy SNP. HPS provided data on 151 directly genotyped SNPs from GWAS and IPLEX experiments, including 48 of the requested SNPs and 103 proxy SNPs (*r*^2^>0.8). Analysis in HPS was not adjusted for ln baseline LDL-C levels. In addition, the number of subjects with data varied from SNP-to-SNP and ranges from ~4,000 for variants with GWAS data to ~18,000 for some candidate genes. Results of the first and second stage were combined using fixed effects, inverse variance weighted meta-analysis and analysed by METAL. As a third stage, six SNPs with *P* values 5 × 10^−8^<*P*<5 × 10^−7^ in the combined meta-analysis were selected for additional genotyping in the Scandinavian participants of the ASCOT study. Kaspar assays were designed for four of the SNPs using the KBioscience Primerpicker software, and oligos were provided by Intergrated DNA technologies ( http://eu.idtdna.com/site). Full Kaspar methodology is available from LGC SNP genotyping ( http://www.lgcgenomics.com/genotyping/kasp-genotyping-reagents/). Two SNPs (rs981844 and rs13166647) were genotyped using Taqman assays supplied by Life Technologies ( http://www.lifetechnologies.com/uk/en/home.html) using the standard Taqman protocol. Results of the additional genotyping were combined with results from the first and second stages using a fixed effects, inverse variance weighted meta-analysis and analysed by METAL.

### Determination of changes in LDL subfractions

LDL subclasses were analysed as described previously^29^ using non-denaturing gradient gel electrophoresis of fasting plasma samples taken at baseline and after 6 weeks of simvastatin 40 mg per day (CAP study, *n*=579) or 12 weeks of pravastatin 40 mg per day (PRINCE study, *n*=1,284). Aliquots of 3.0 ml of whole plasma were mixed 1:1 with a sampling buffer of 20% sucrose and 0.25% bromophenol blue. Electrophoresis of samples and size calibration standards was performed using 2–14% polyacrylamide gradients at 150 V for 3 h following a 15-min pre-run at 75 V. Gels were stained with 0.07% Sudan black for 1 h and stored in a 0.81% acetic acid, 4% methanol solution until they were scanned by computer-assisted densitometry for determination of areas of LDL IVb (22.0–23.2 nm), LDL IVa (23.3–24.1 nm), LDL IIIb (24.2–24.6 nm), LDL IIIa (24.7–25.5 nm), LDL IIb (25.6–26.4 nm), LDL IIa (26.5–27.1 nm) and LDL I (27.2–28.5 nm). The cholesterol concentrations of the subfractions (mg dl^−1^ plasma) were determined by multiplying percent of the total stained LDL area for each subfraction by the LDL-C for that sample. For genetic association analyses, subfractions were grouped into large LDL (LDL I+IIa), medium LDL (LDL IIb), small LDL (LDL IIIa) and very small LDL (LDL IIIb+IVa+IVb) as described previously^18^. A generalized estimating equation method was used to test the association of log change with the interaction of the four SNPs by LDL subfraction.

### Effect of off-treatment LDL-C

Effects of genetic variation on treatment response as measured by on-treatment LDL-C could be mediated through effects on the off-treatment LDL-C. To evaluate whether genetic on-treatment LDL-C likely reflects residual effect on off-treatment LDL-C, it is necessary to adjust for the off-treatment LDL-C levels and to correct the maximum likelihood estimate of the adjusted effect of genotype on on-treatment value for the noise in off-treatment values (the noise is both random measurement error and intra-individual variation in usual LDL-C). This analysis was only carried out in CARDS in which multiple baseline measurements were available. From the rules of path analysis, we calculated the direct effect *γ* of genotype on an on-treatment trait value as *β*−*αδ* (1−*ρ*)/*ρ*, where *β* is the coefficient of regression for on-treatment trait value on genotype adjusted for measured off-treatment value, *α* is the coefficient of regression of baseline LDL on genotype, *ρ* is the intraclass correlation between replicate measurements of off-treatment values and *δ* is the coefficient of regression for on-treatment value on observed off-treatment value^8^. For these calculations, we used *ρ*=0.8 as a plausible value for the intraclass correlation based on the within-person correlation in LDL-C values taken over two off-treatment visits in CARDS. The interaction of candidate SNPs with statin versus placebo allocation was assessed in the JUPITER trial, since this study was not involved in the first-stage meta-analysis. Regression models were applied to the combined population of statin- and placebo-treated subjects by including extra terms encoding placebo allocation and the product of placebo allocation with SNP minor allele dose^7^.

### GWCA using Genome-Complex Trait Analysis

There may be multiple causal variants in a gene and the total variation that could be explained at a locus may be underestimated if only the most significant SNP in the region is selected. To identify independent SNPs, we ideally can perform a conditional analysis, starting with the top associated SNP, across the whole genome followed by a stepwise procedure of selecting additional SNPs, one by one, according to their conditional *P* values. Such a strategy would allow the discovery of more than two associated SNPs at a locus. To identify independent SNPs across the genome-wide data, we used an approximate conditional and joint analysis approach implemented in Genome-Complex Trait Analysis (GCTA) software ( http://www.complextraitgenomics.com/software/gcta/). We used summary-level statistics from the first- and second-stage-combined meta-analysis and LD corrections between SNPs estimated from CARDS GWAS data. SNPs on different chromosomes or more than 10 Mb distant are assumed to be in linkage equilibrium. The model selection process in GCTA starts with the most significant SNP in the single-SNP meta-analysis across the whole genome with *P* value <5 × 10^−7^. In the next step, it calculates the *P* values of all the remaining SNPs conditional on the top SNP that have already been selected in the model. To avoid problems due to colinearity, if the squared multiple correlations between a SNP to be tested and the selected SNP(s) is larger than a cut-off value, such as 0.9, the conditional *P* value for that SNP will be set to 1. Select the SNPs with minimum conditional *P* value that is lower than the cut-off *P* value. Fit all the selected SNPs jointly in a model and drop the SNPs with the *P* value that is greater than the cut-off *P* value. This process is repeated until no SNPs can be added or removed from the model.

### Pathway analysis and construction of a statin response network

Genes showing evidence of association (based on direct association or LD (HapMap CEU *r*^2^>0.8)) were reviewed for evidence of involvement in statin response at a pathway level using GeneGo Metacore (Thomson Reuters (portal.genego.com)). A statin response network was constructed in two stages. First, all genes with a literature-reported involvement in statin response (based on Medical Subject Headings (MeSH)) were identified using GeneGo MetaCore (Supplementary Data 3). Second, these genes were combined with all genes in associated loci (including genes in LD) and a network was constructed based on direct interactions only. By including direct interactions only, we created a conservative network of direct gene interactions that have been consistently linked to statin response in the literature.

### eQTL analysis

LDL-C-associated index SNPs (246 SNPs) were used to identify 1,443 LD proxy SNPs displaying complete LD (*r*^2^=1) across four HapMap builds in European ancestry samples (CEU) using the SNAP tool ( http://www.broadinstitute.org/mpg/snap/). The primary index SNPs and LD proxies were searched against a collected database of expression SNP (eSNP) results, including the following tissues: fresh lymphocytes^30^, fresh leukocytes^31^, leukocyte samples in individuals with Celiac disease^32^, whole-blood samples^33^,^34^,^35^,^36^, lymphoblastoid cell lines (LCL) derived from asthmatic children^37^,^38^, HapMap LCL from three populations^39^, a separate study on HapMap CEU LCL^40^, additional LCL population samples^41^,^42^,^43^ (Mangravite *et al.*, unpublished), CD19+ B cells^44^, primary phytohaemagglutinin-stimulated T cells^41^, CD4+ T cells^45^, peripheral blood monocytes^44^,^46^,^47^, CD11+ dendritic cells before and after *Mycobacterium* tuberculosis infection^48^, omental and subcutaneous adipose^33^,^43^,^49^, stomach^49^, endometrial carcinomas^50^, ER+ and ER− breast cancer tumour cells^51^, brain cortex^46^,^52^,^53^, prefrontal cortex^54^,^55^, frontal cortex^56^, temporal cortex^53^,^56^, pons^56^, cerebellum^53^,^56^, three additional large studies of brain regions including prefrontal cortex, visual cortex and cerebellum, respectively^57^, liver^49^,^58^,^59^, osteoblasts^60^, ileum^49^,^61^, lung^62^, skin^43^,^63^ and primary fibroblasts^41^. Micro-RNA QTLs were also queried for LCL^64^ and gluteal and abdominal adipose^65^. The collected eSNP results met the criteria for association with gene expression levels as defined in the original papers. In each case where a LDL-C-associated SNP or proxy was associated with a transcript, we further examined the strongest eSNP for that transcript within that data set (best eSNP), and the LD between the best eSNP and GIST-selected eSNPs to estimate the concordance of the LDL-C and expression signals.

### Statin response connectivity map analysis

The Connectivity Map (Cmap) data set is available at the Broad Institute ( www.broadinstitute.org/cmap) and contains more than 7,000 expression profiles representing 1,309 compounds used on five different cultured human cancer cell lines (MCF7, ssMCF7, HL60, PC3 and SKMEL5). We selected (prostate tumour-derived) PC3 cells as they showed the most responsiveness to statins at a genome-wide level. Four statins were included in our analysis, including pravastatin, atorvastatin, simvastatin and rosuvastatin. PC3 Instance reference files for each statin treatment were extracted (as defined by Lamb *et al.*^12^), that is, a treatment associated to its control pair. Transcripts were considered to show evidence of differential expression with a fold change >2. A fold change >1.5 was considered to be suggestive of differential expression only.

### Exploration of functional impact among directly and indirectly associated variants

Genes and variants across all LDL-C-associated loci were investigated for evidence of functional perturbation using a range of bioinformatics tools and databases. Variants showing LD (CEU *r*^2^>0.8) with associated variants were explored for impact on coding gene function using Annovar^66^ and regulatory function using a combination of HaploReg^67^ and Regulomedb^68^, which both draw on comprehensive data from the Encyclopedia of DNA Elements (ENCODE)^69^ and the NIH Roadmap Epigenomics consortium^70^. Building on the functional annotation, we also identified variants that were shown to mediate eQTLs. Genes in associated loci were also used to query the NIH connectivity map for evidence of differential expression in PC3 cell lines treated with pravastatin, simvastatin and rosuvastatin. By combining a wide range of functional data and pathway support, we were able to build up a view of genes with the highest level of support in statin response.

## Author contributions

I.P., S.T., H.A.D., M.R.B., X.L., H.R.W., D.I.C., K.Z., B.J.A., B.M.P., G.H., R.M.K., J.W.J. and M.J.C. constituted the writing and analysis group. I.P., S.T., H.A.D. and K.Z. performed quality control on the individual study summary results. I.P. and S.T. performed meta-analysis. I.P., H.A.D., M.R.B., X.L., H.R.W., D.I.C. and R.M.K. performed additional analyses. All analysis and writing group authors extensively discussed the analysis, results, interpretation and presentation of results. All authors contributed to the research and reviewed the manuscript.

Study concept and design of contributing studies by (PROSPER) J.W.J., D.J.S., B.M.B., I.F., N.S. and R.G.J.W.; (ASCOT) M.J.C., P.S., N.P., A.S., D.C.S. and E.O.; (CARDS) H.A.D., H.M.C., P.M.M., J.B., P.N.D., A.D. and G.H.; (PARC) X.L., Y.-D.I.C., J.I.R. and R.M.K.; (TNT) J.J.P.K.; (AGES) L.J.L., T.B.H. and V.G.; (ARIC) C.L.A., E.A.W., T.S., E.B. and C.M.B.; (BioVU) Q.F., W.-Q.W., R.A.W. and J.C.D.; (CHS, HVH) N.S., K.R., T.L., J.I.R., B.M.P. and S.R.H.; (FHS) L.A.C. and V.R.; (GoDARTS) C.N.A.P. and H.M.C.; (HABC) Y.L.; (MESA) X.G., S.R.H., W.P. and J.I.R.; (Rotterdam Study) C.E.d.K., B.H.S., A.G.U., A.H. and F.R.; and (JUPITER) D.I.C., B.J.B., F.N. and P.M.R.

Phenotype data acquisition of contributing studies by (PROSPER) J.W.J., D.J.S., B.M.B., I.F., A.J.M.d.C., N.S. and R.G.J.W.; (ASCOT) M.J.C., P.B.M., P.S., N.P., A.S., D.C.S., E.O. and S.S.H.; (CARDS) H.A.D., H.M.C., P.M.M., J.B., P.N.D., A.D. and G.H.; (PARC) X.L., Y.-D.I.C., J.I.R. and R.M.K.; (TNT) J.J.P.K.; (AGES) G.E.; (ARIC) C.M.B.; (BioVU) W.W.; (CHS, HVH) K.L.W., J.C.B., A.M.A., N.L.S., B.M.P. and S.R.H.; (FHS) L.A.C., C.J.O., V.R.; (GoDARTS) C.N.A.P., L.A.D., K.Z., A.D., A.M. and H.M.C.; (HABC) D.M.H. and S.B.K.; (MESA) W.P. and J.I.R.; (Rotterdam Study) C.E.K., B.H.S., A.H. and O.H.F.; (JUPITER) D.I.C., F.G., J.G.M. and P.M.R.

Genotype data acquisition of contributing studies by (PROSPER) S.T., J.W.J., A.J.M.C. and P.E.S.; (ASCOT) M.J.C., P.B.M., P.S., A.S. and S.S.H.; (CARDS) H.A.D., H.M.C., P.M.M., P.N.D., A.D. and G.H.; (PARC) Y.-D.I.C., J.I.R., D.A.N. and J.D.S.; (TNT) B.J.A., M.P.D., S.M.B., G.K.H. and J.-C.T.; (AGES) A.V.S.; (ARIC) E.B.; (BioVU) Q.F., J.C.D., C.T.L. and F.S.; (CHS, HVH) G.L., J.C.B., K.D.T., J.I.R., K.R., T.L. and S.R.H.; (FHS) C.J.O.; (GoDARTS) C.N.A.P., K.Z., A.D., F.C., H.M.C., M.I.M., L.G., E.A. and WTCCC2; (HABC) Y.L.; (MESA) K.D.T. and J.I.R.; (Rotterdam Study) A.G.U. and F.R.; and (JUPITER) D.I.C., F.G., B.J.B., F.N. and P.M.R.

Primary analysis from contributing studies by (PROSPER) I.P., S.T., A.J.M.C. and P.E.S.; (ASCOT) M.J.C., M.R.B. and H.R.W.; (CARDS) H.A.D., H.M.C. and P.M.M.; (PARC) X.L., Y.-D.I.C. and J.I.R.; (TNT) B.J.A., M.P.D., S.M.B., G.K.H. and J.-C.T.; (AGES) A.V.S.; (ARIC) C.L.A., E.A.W. and T.S.; (BioVU) Q.F., W.W., C.T.L. and F.S.; (CHS, HVH) K.L.W. and G.L.; (FHS) L.A.C., P.G. and J.S.N.; (GoDARTS) C.N.A.P., L.A.D., K.Z. and H.M.C.; (HABC) D.S.E., J.M.S. and J.D.; (MESA) K.D.T., X.G., X.L. and J.I.R.; (Rotterdam Study) C.E.K. and B.H.S.; and (JUPITER) D.I.C., A.Y.C., F.G., J.G.M. and P.M.R.

## Additional information

**How to cite this article:** Postmus, I. *et al.* Pharmacogenetic meta-analysis of genome-wide association studies of LDL cholesterol response to statins. *Nat. Commun.* 5:5068 doi: 10.1038/ncomms6068 (2014).

## Supplementary Material

Supplementary Figures, Supplementary Tables, Supplementary Notes and Supplementary References Supplementary Figures 1-4, Supplementary Tables 1-10, Supplementary Notes 1-3 and Supplementary References

Supplementary Data 1 Expression Quantitative trait loci (eQTLs) linked to LDL-C associated SNPS

Supplementary Data 2 Functional characterization of all variants showing LD with with LDL-C associated SNPs

Supplementary Data 3 Candidate loci defined by association and eQTLs identified in this study

Supplementary Data 4 GeneGo custom network of known statin interacting genes

## Figures and Tables

**Table 1:** Genome-wide significant associations in stage 1, stage 2 and combined meta-analysis.

**Chr**	**Position**	**Lead SNP**	**Gene**	**Coding allele**	**Noncoding allele**	**Phase**	***N***	**Frequency-coding allele**	Beta*	**s.e.**	**% Extra reduction**†	***P*** **value**
1	109620053	rs646776	*SORT1/CELSR2/PSRC1*	C	T	Stage 1	16,697	0.230	−0.015	0.003	1.5	6.70 × 10^−7^
						Stage 2	21,902	0.216	−0.010	0.003	1.0	2.43 × 10^−4^
						Combined	38,599		−0.013	0.002	1.3	1.05 × 10^−9^
6	160930108	rs10455872	*LPA*	G	A	Stage 1	12,981	0.069	0.041	0.006	−4.1	1.95 × 10^−11^
						Stage 2	18,075	0.087	0.059	0.005	−5.9	7.14 × 10^−35^
						Combined	31,056		0.052	0.004	−5.2	7.41 × 10^−44^
12	21260064	rs2900478	*SLCO1B1*	A	T	Stage 1	16,749	0.165	0.016	0.003	−1.6	2.26 × 10^−6^
						Stage 2	7,504	0.164	0.017	0.006	−1.7	3.54 × 10^−3^
						Combined	24,253		0.016	0.003	−1.6	1.22 × 10^−9^
19	50107480	rs445925	*APOE*	A	G	Stage 1	13,909	0.098	−0.043	0.005	4.3	1.58 × 10^−18^
						Stage 2	3,613	0.157	−0.088	0.011	8.8	1.41 × 10^−15^
						Combined	17,522		−0.051	0.005	5.1	8.52 × 10^−29^

Chr, chromosome; SNP, single nucleotide polymorphism.

^*^Beta for difference between the natural log-transformed on- and off-treatment low-density lipoprotein cholesterol (LDL-C) levels adjusted for natural log-transformed off-treatment LDL-C-, age-, sex- and study-specific covariates. The beta reflects the fraction of differential LDL-C lowering in carriers versus non-carriers of the SNP; a negative beta indicates a better statin response (stronger LDL-C reduction), a positive beta a worse statin response. Betas and *P* values were generated using linear regression analysis.

^†^This percentage reflects the % extra LDL-C lowering in carriers versus non-carriers of the SNP.

**Table 2:** Associations of the minor alleles of rs646776, rs445925, rs2900478 and rs10455872 with changes in LDL-C and LDL subfractions in response to statin in the combined CAP and PRINCE studies.

Change*	***SORT1/CELSR2/PSRC1*****rs646776 (MAF 0.2)**			***APOE*** **rs445925 (MAF 0.086)**			***SLCO1B1*** **rs2900478 (MAF 0.16)**			***LPA*** **rs10455872 (MAF 0.056)**		
	**Beta**	**s.e.**	***P*** **value**	**Beta**	**s.e.**	***P*** **value**	**Beta**	**s.e.**	***P*** **value**	**Beta**	**s.e.**	***P*** **value**
LDL-C total	−0.023	0.008	0.003	−0.046	0.018	0.008	0.010	0.005	0.04	0.032	0.019	0.09
Large LDL-C	−0.028	0.014	0.042	−0.075	0.029	0.009	0.02	0.008	0.01	0.036	0.031	0.23
Medium LDL-C	−0.027	0.015	0.075	−0.079	0.032	0.012	0.016	0.009	0.07	0.010	0.034	0.77
Small LDL-C	−0.047	0.018	0.009	−0.071	0.037	0.050	0.002	0.010	0.83	−0.024	0.039	0.54
Very small LDL-C	−0.034	0.009	0.00006	−0.022	0.017	0.202	0.001	0.005	0.90	0.008	0.019	0.67

LDL-C, low-density lipoprotein cholesterol; MAF, minor allele frequency.

^*^Change: ln (on treatment)−ln (baseline) models adjusted for log (baseline variable), age, sex, body mass index, smoking(y/n) and study (CAP versus PRINCE). Betas and *P* values were assessed using a generalized estimating equation method.
