# Supplementary material for: Pharmacogenetic meta-analysis of genome-wide association studies of LDL cholesterol response to statins
Source: Nat Commun. 2014 Oct 28;5:5068. doi: 10.1038/ncomms6068 (PMC4220464; doi:10.1038/ncomms6068)
Supplement: Supplementary Figures, Supplementary Tables, Supplementary Notes and Supplementary References — Supplementary Figures 1-4, Supplementary Tables 1-10, Supplementary Notes 1-3 and Supplementary References [file ncomms6068-s1.pdf]

Supplementary figures

Supplementary Figure 1: Quantile-quantile plots of the expected versus observed -logP values for all studies participating in the first stage meta-analysis. P-values were generated using linear regression analysis.

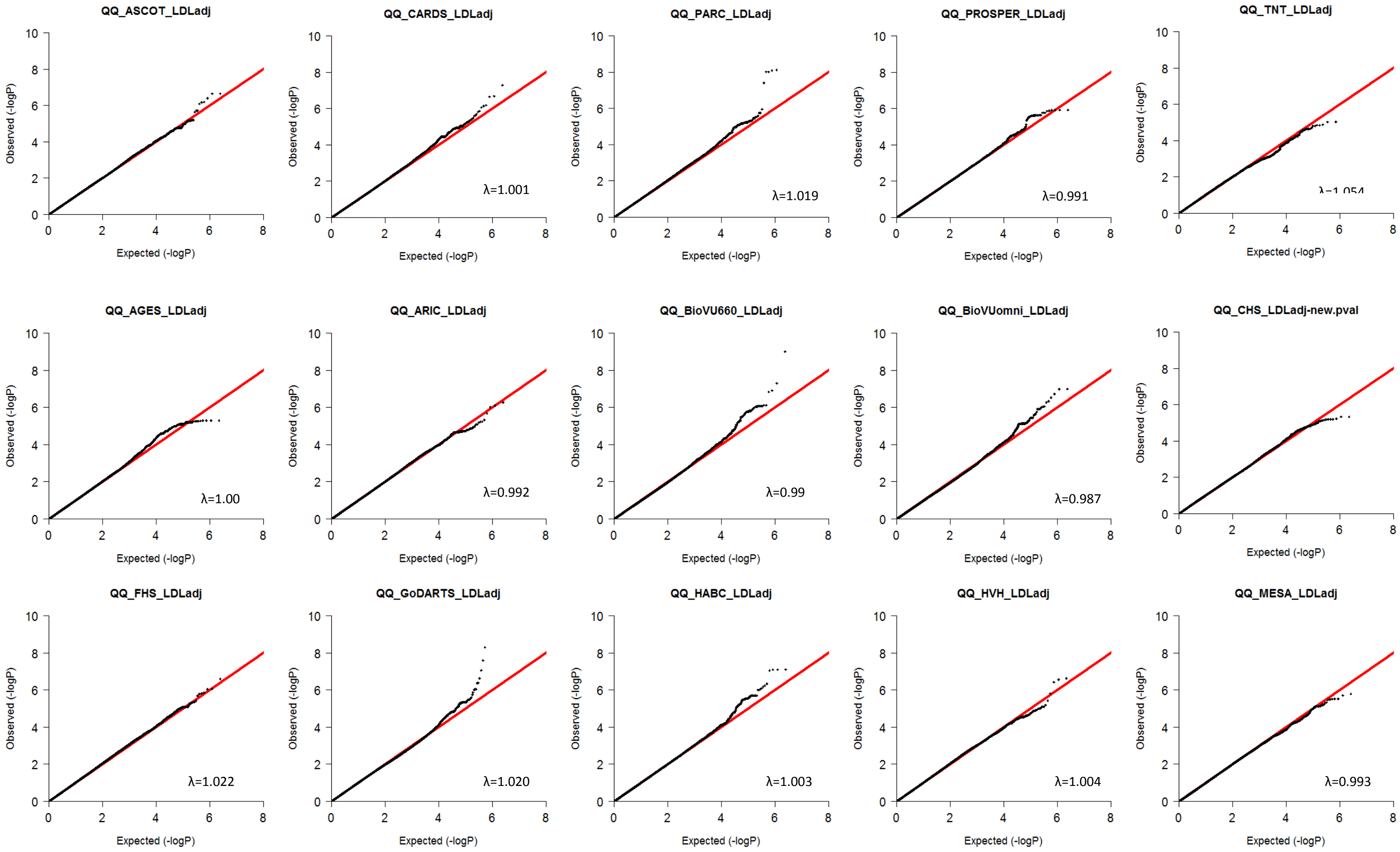

Supplementary Figure 2: Forest plots showing the association between the four genome-wide significant SNPs in the combined meta-analysis and LDL-C response to statin treatment in all individual studies (n=38,599). A negative beta indicates a better statin response (stronger LDL-C reduction), a positive beta a worse statin response. Betas were assessed using linear regression analysis and error bars reflect the 95% confidence interval.

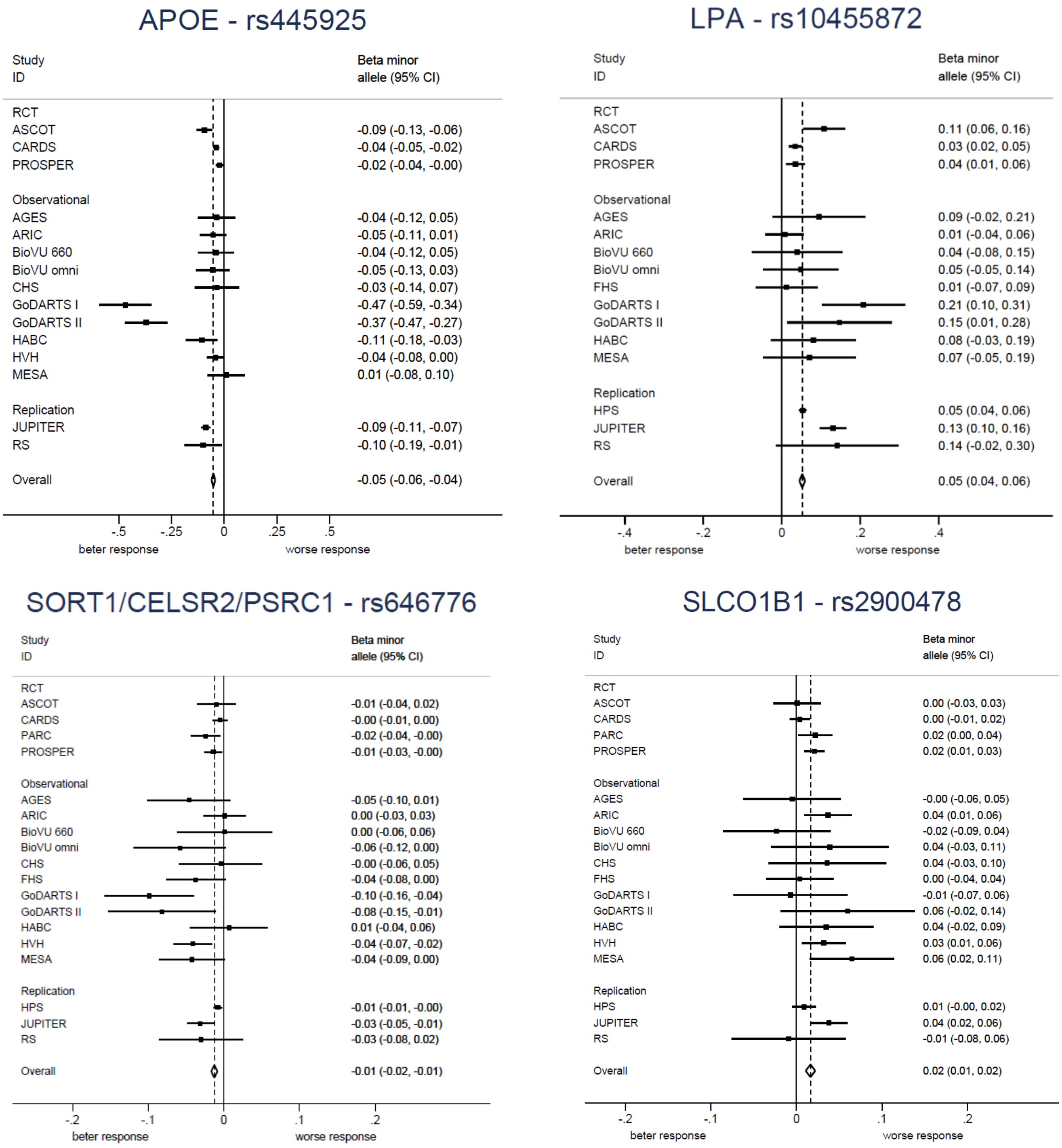

Supplementary Figure 3: Percent change (mean ± SEM) in concentrations of LDL subfractions (mg/dL cholesterol) measured before and after simvastatin 40 mg/d for 6 weeks (CAP study, n=579) or pravastatin 40 mg/d for 12 weeks (PRINCE study, n=1284). Subfractions ranging in particle size from largest (LDL I) to smallest (LDL IVb) were analyzed by gradient gel electrophoresis as described in Methods.

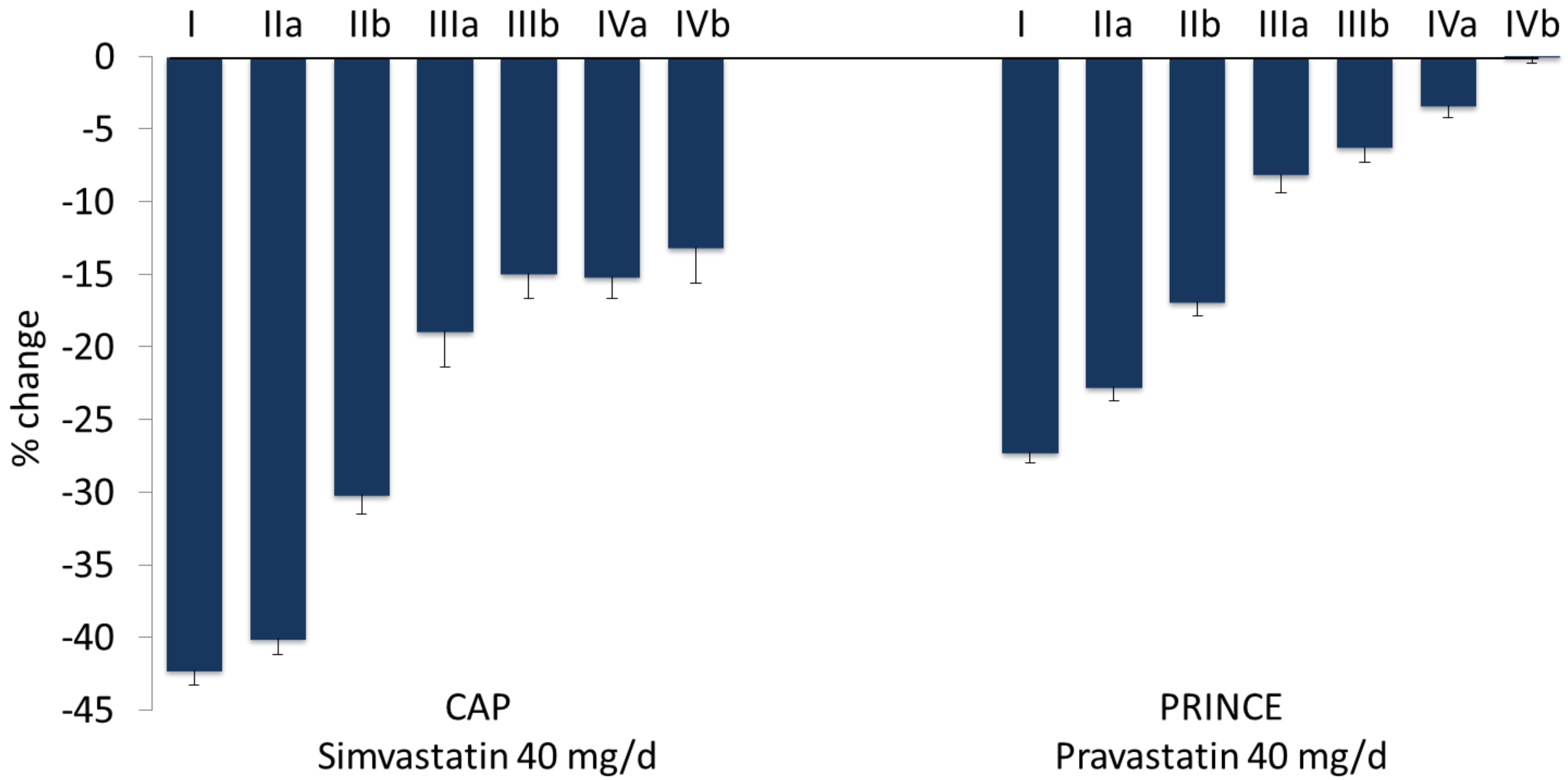

Supplementary figure 4: Core gene interaction network between GIST GWAS associated genes and known statin interacting genes. GWAS associated genes reported in this study are indicated by a blue dot, the minimum meta-analysis p value is indicated, alongside the total number of network interactions . Gene products with a known interaction with the statin drug class were identified by Medline subject headings (MESH) and by query of the GeneGO metacore interaction database (Thomson Reuters). The network was prepared using the custom network feature in GeneGo metacore.

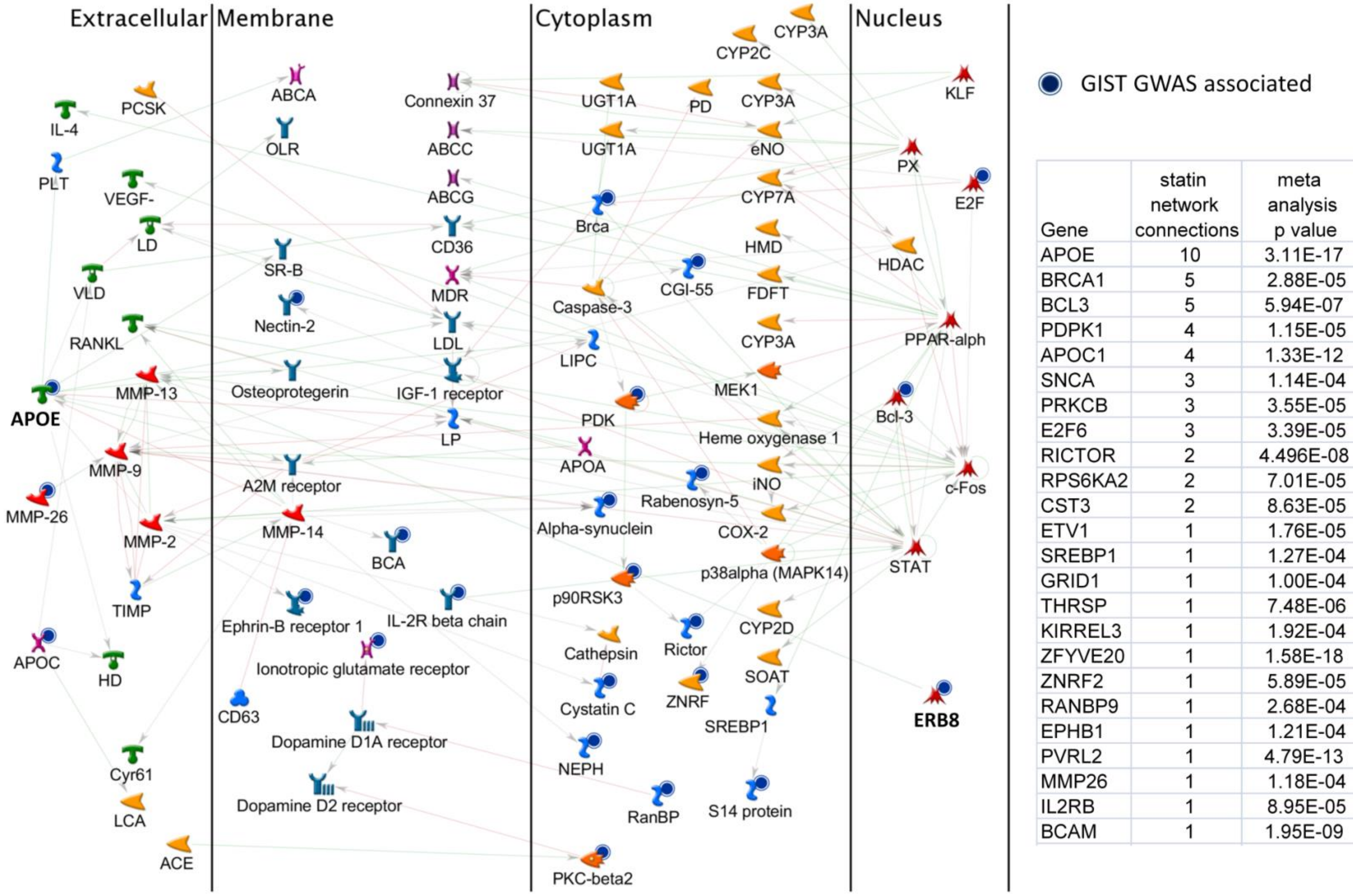

Gene	statin network connections	meta analysis p value
APOE	10	3.11E-17
BRCA1	5	2.88E-05
BCL3	5	5.94E-07
PDPK1	4	1.15E-05
APOC1	4	1.33E-12
SNCA	3	1.14E-04
PRKCB	3	3.55E-05
E2F6	3	3.39E-05
RICTOR	2	4.496E-08
RPS6KA2	2	7.01E-05
CST3	2	8.63E-05
ETV1	1	1.76E-05
SREBP1	1	1.27E-04
GRID1	1	1.00E-04
THRSP	1	7.48E-06
KIRREL3	1	1.92E-04
ZFYVE20	1	1.58E-18
ZNRF2	1	5.89E-05
RANBP9	1	2.68E-04
EPHB1	1	1.21E-04
PVRL2	1	4.79E-13
MMP26	1	1.18E-04
IL2RB	1	8.95E-05
BCAM	1	1.95E-09

Supplementary Table 1: Participating study characteristics of the statin users only.

Study sample	Participants	Male, N (%)	Age*, mean ± SD	Age*, range	Body mass index, kg/m^2, mean ± SD	Diabetes, N (%)	Hypertension, N (%)
**Randomized controlled trials**	***N Overall***						
PROSPER	2550	1228 (48)	75.4 (3.4)	70.2-83.3	26.8 (4.1)	256 (10.0)	1592 (62.4)
ASCOT UK	913	807 (88)	63.7 (8.1)	40-79	29.0 (5.1)	192 (21.0)	913 (100)
CARDS	1194	632 (53)	61.6 (8.2)	40-76	28.7 (3.6)	1194 (100)	1038 (87)
PRINCE	1348	1040 (77)	64.8 (13.0)	26-100	29.0 (5.3)	271 (20.1)	551 (40.9)
CAP	591	312 (53)	54.5 (12.6)	30-88	27.7 (5.5)	21 (3.6)	108 (18.3)
TNT	1845	1521 (82)	62.4 (8.4)	36.4-76.0	29.1 (4.7)	411 (22.3)	631 (34.2)
**Observational studies**	***N Overall***						
AGES	280	122 (44)	74.4 (4.8)	66-92	27.5 (4.1)	58 (20.7)	236 (84.3)
ARIC	1067	545 (51)	58.9 (5.8)	45-71	27.5 (4.6)	140 (13.1)	390 (36.8)
BioVu	556	309 (56)	55.7 (14.4)	15-92	29.2 (6.0)	307 (55.2)	550 (98.9)
CHS	312	115 (37)	69.5 (3.1)**	65-87	26.6 (4.2)	23 (7.4)	91 (29.2)
FHS	669	376 (56)	59.4 (8.4)	32-85	28.5 (4.8)	109 (16.3)	357 (53.3)
GODARTS I	3404	1830 (54)	65.4 (10.7)	58.6-73.0	30.6 (6.1)	3404 (100)	3271 (96.0)
GODARTS II	2377	1335 (56)	56.9 (11.4)	58.9-74.0	31.3 (6.1)	2377 (100)	2266 (95.3)
Health ABC	294	164 (56)	73.4 (2.7)	69-80	27.1 (4.2)	53 (21.1)	198 (67.4)
HVH	1518	638 (42)	68.5 (9.3)**	34-90	31.1 (6.7)	374 (24.7)	1156 (76.2)
MESA	360	180 (50)	66.9 (9.3)	47-87	28.9 (5.4)	48 (13.4)	191 (53.1)
**Second stage studies**	***N Overall***						
HPS	18705	14033 (75)	64.1 (8.35)	40-82	27.6 (4.4)	5278 (28.2)	7720 (41.3)
JUPITER	3401	2332 (69)	66.0 (7.6)	50-93	29.5 (5.8)	12 (0.3)	1892 (55.6)
Rotterdam Study	212	105 (45)	61.4 (4.0)	55.2-72.4	27.8 (4.0)	38 (17.9)	116 (54.7)
**Additional genotyping**	***N Overall***						
ASCOT Scandinavians***	1411	1116 (79)	60.8 (8.6)	40-80	28.7 (4.8)	310 (22)	1411 (100)

*Age at DNA collection

**Age at baseline

***Combined sample of RCT and observational trial arm

Supplementary Table 2: Low-density lipoprotein characteristics of the statin users only.

Study sample	Participants	Type of statin	Statin dose (mg/day)	LDL-C off-treatment (mmol/L) Mean ± SD	LDL-C on-treatment (mmol/L)* Mean ± SD	Follow-up time (months) Mean ± SD
**RCTs**	***N Overall***					
PROSPER	2550	Pravastatin	40	3.80 (0.81)	2.57 (0.66)	29.5 (9.2)
ASCOT UK	913	Atorvastatin	10	3.46 (0.71)	2.19 (0.63)	First year used
CARDS	1194	Atorvastatin	10	3.04 (0.71)	2.11 (0.70)	46.8 (4.7)
PRINCE	1348	Pravastatin	40	3.37 (0.75)	2.50 (0.68)	12 weeks
CAP	591	Simvastatin	40	3.41 (0.81)	1.97 (0.61)	6 weeks
TNT	1845	Atorvastatin	10	4.18 (0.59)	2.52 (0.41)	2.0 (0.2)
**Observational studies**	***N Overall***					
AGES	280	mixed	mixed	4.17 (0.97)	2.18 (0.63)	62.4 (4.8)
ARIC	1067	A, Ce, F, L, P, S, Ch	Not available	4.27 (0.98)	3.08 (0.78)	36.1 (3.14)
BioVu	556	A, S, F, P, L, R	5,10,20,40,80	5.83 (1.40)	4.52 (1.00)	12.1 (6.4)
CHS	312	A, P, L, S, F, Ce	14.1 (8.5), 20.8 (9.3), 21.4 (8.9), 16.5 (10.6), 35.0 (23.7), 0.37 (0.08)	3.88 (1.02)	2.33 (1.97)	43.3 (43.2)
FHS	669	mixed	mixed	3.91 (0.94)	2.68 (0.80)	52.8 (21.6)
GODARTS I	3205	mixed	mixed	3.61 (0.96)	1.60 (0.69)	51.0 (37.9)
GODARTS II	2158	mixed	mixed	3.51 (0.95)	1.63 (0.70)	47.7 (38.9)
Health ABC	294	mixed	mixed	3.55 (0.86)	2.26 (0.62)	50.9 (17.4)
HVH	1518	A, P, L, S, R	34.6 (24.0), 20.8 (4.9), 33.5 (9.8), 36.7 (15.2), 20.0	3.83 (1.10)	2.50 (0.85)	4.0 (6.1)
MESA	360	mixed	mixed	3.44 (0.77)	2.24 (0.57)	19.9 (3.2)
**Second stage studies**	***N Overall***					
HPS	18705	Simvastatin	40	3.37 (0.81)	1.98 (0.64)	1.90 (0.24)
JUPITER	3401	Rosuvastatin	20	2.75 (0.45)	1.42 (0.54)	12 months
Rotterdam Study	212	S (N=129), P(N=17), F(N=7), A(N=48), (N=11)	24.1 (11.6), 31.2 (11.1), 65.7 (25.1), 17.5 (9.8), 7.7 (4.7)	4.13 (0.90)	2.26 (0.69)	210.0 (6.2)[#] 133.2 (4.6)[$]
**Additional genotyping**	***N Overall***					
ASCOT Scan RCT	748	Atorvastatin	10	3.43 (0.70)	2.24 (0.63)	9.0 (1.2)
ASCOT Scan Obs	663	A (N=547), F (N=7), L (N=1), P (N=14), R (N=10), S (N=84)	11.8 (4.5), 60.0 (25.8), 20.0, 31.4 (10.3), 10.0, 21.5 (10.3)	3.89 (1.00)	2.51 (0.76)	16.6 (6.9)

*Mean of multiple on-treatment measurements

[#] Mean time between start RS and LDL-measurement on treatment, [$] Mean time between off-treatment LDL and on-treatment LDL measurement

Abbreviations: A, Atorvastatin; Ce, Cerivastatin; F, Fluvastatin; L, Lovastatin; P, Pravastatin; S, Simvastatin; Ch, Cholestin; R, Rosuvastatin

Supplementary Table 3: Statin dose adjustments in observational studies, based on a modified version of a table in Drugs 1998; 56: Suppl 1: 25-31[1].

Statin	Dose range (mg)	Typical starting dose (PDR)	Dose % equivalent	Reduction LDL (%)	Reduction TC (%)
Atorvastatin	10-80	10-20	10	35	29
Cerivastatin	0.2-0.4	0.2-0.3	0.3	30	-
Fluvastatin	10-80	40	60	31	23
Lovastatin	10-80	20	40	32	23
Pravastatin	10-40	40	40	30	25
Simvastatin	10-80	20-40	20	36	28
Rosuvastatin	5-40	10	5	45	33
Pitavastatin	2-4	2	2	37	-

Supplementary Table 4: Genotyping characteristics

Study sample	Participants	Genotyping platform	Calling algorithm	NCBI build	Imputation software	Analysis software	Exclusion criteria used
**RCTs**	***N Overall***						
PROSPER	2550	Illumina Human 660_Quadv1	Beadstudio	36.22	MACH v1.0 16	ProbABEL	Sample call rate>=97.5%, SNP call rate >=98%, SNP MAF>0.01
ASCOT UK	913	Illumina Human 370CNV	Beadstudio	36.22	MACH v1.0 16	ProbABEL	Sample call rate <=95%, SNP call rate <=97%, HWE<=10E-7, relatedness
CARDS	1194	Perlegen 6	Perlegen 6	36.22	Impute2	SNPTEST	Sample call rate>=98%<br>SNP call rate>=98%<br>MAF>0.01
PRINCE	1348	Illumina Human 317K and 610_Quad	Illumina	36.23	Bimbam v0.99	SNPTEST	Imputation information>0.30, SNP MAF>0.01
CAP	591	Illumina Human 317K and 610_Quad	Illumina	36.23	Bimbam v0.99	SNPTEST	Imputation information>0.30, SNP MAF>0.01
TNT	1845	Perlegen 322K	Perlegen	36.3	IMPUTE 2 v2.1.0, GTOOL v 0.6.6	Plink v 1.07	Sample call rate>=98%,<br>SNP call rate >=98%
**Observational**	***N Overall***						
AGES	280	Illumina HU370CNV	Illumina Beadstudio	36	MACH v1.0 16	ProbABEL	Pre imputation exclusions: MAF >0.01, HWE p 10-6, callrate 0.97. Call rate 0.95
ARIC	1067	Affymetrix 6.0	Birdseed	36	MACH v1.0.16	ProbABEL	MAF <1%, call rate <95%, HWE<10E-5
BioVu	556	Illumina 660K, Illumina OMNI	Illumina	36(660K), 37.1(OMNI)	MACH v1.0.16	R	Sample call rate>=98%, SNP call rate >=98%, SNP MAF>0.01, Analysis of these clinical practice-based data limited to subjects with delta LDL-C available for their "1st" statin
CHS	312	Illumina Human 370CNV	BeadStudio	36	BIMBAM	R	Samples excluded for sex mismatch, discordance with prior genotyping, or call rate < 95%<br>SNPs excluded for: call rate < 97%, HWE P < 10-5, > 2 duplicate errors or Mendelian inconsistencies (for reference CEPH trios), heterozygote frequency = 0, SNP not found in HapMap.

Study sample	Participants	Genotyping platform	Calling algorithm	NCBI build	Imputation software	Analysis software	Exclusion criteria used
FHS	669	Affymetrix 250K Sty, 250K Nsp & MIPS 50K Gene Centric	BRLMM	HapMap 36.22	MACH v1.0.15	R 2.6.1 with lmekin	Sample call rate ≤ 97%, SNP call rate ≤ 95%, SNP >1000 Mendelian errors, Heterozygosity 5 SD from Mean (<25.758% or >29.958%)
GODARTS I	3205	Affymatrix 6.0	CHIAMO	36.3	IMPUTE2	SNP TEST	Sample call rate<=98%, SNP call rate <=98%, SNP MAF<0.01; p-HWE<1^{e-6}
GODARTS II	2158	HumanOmniExpress	Illumina	36.3	IMPUTE2	SNP TEST	Sample call rate<=98%, SNP call rate <=98%, SNP MAF<0.01; p-HWE<1^{e-6}
Health ABC	294	Illumina Human1M-Duo BeadChip	Illumina BeadStudio	release 22, build 36	MACH v1.0.16	R	Sample call rate>=97%, SNP call rate >=97%, SNP MAF>0.01
HVH	1518	Illumina Human 370CNV	BeadStudio	36	BIMBAM	R	Samples excluded for sex mismatch or call rate < 95%. SNP exclusions: call rate < 97%, HWE P < 10^{-5}, > 2 duplicate errors or Mendelian inconsistencies (for reference CEPH trios), heterozygote frequency = 0, SNP not found in HapMap, inconsistencies across genotyping batches.
MESA	360	Affymetrix Genome-Wide Human SNP Array 6.0	Affymetrix	36.24	IMPUTE v2.1.0	SNPTEST	SNP call rate >=95%, Imputation information>0.30, SNP MAF>0.01
**Second stage**							
HPS	18705 (3895 GWAS)	Illumina 610k Quad and I.PLEX	Beadstudio	36	NA	PLINK/SAS	GWAS: Exclude samples with: Discrepant sex, repeat samples, <95% genotyping success rate. Excluded SNPs with: <0.5% MAF, <95% call rate, HWE p<$5x10^{-7}$
JUPITER	3401	Illumina Omni Quad 1M	Illumina GenomeStudio (v1.6.2)	36	MACH v1.0.16	R	Sample call rate < 90%, SNP call rate <98%
Rotterdam Study	212	Illumina HumanHap 550K	GenomeStudio (Illumina)	36.22	MACH v1.0.15	ProbABEL	Call rate <98%, HWE P<10^{-6}, or MAF<1%

Supplementary Table 5: First stage, second stage, and combined results of all SNPs investigated in the second stage.

					First stage (n=18569)					Second stage (n=22318)					Combined (n=40887)				
SNPID	CHR	position	coded_all	noncoded_all	AF_coded_all	Effect*	SE	P-value	N	AF_coded_all	Effect*	SE	P-value	N	AF_coded_all	Effect*	SE	P-value	N
rs10455872	6	160,930,108	a	g	0.931	-0.041	0.006	1.95E-11	12963	0.913	-0.059	0.005	7.14E-35	18075	0.920	-0.052	0.004	7.41E-44	31038
rs445925	19	50,107,480	a	g	0.098	-0.043	0.005	1.58E-18	14129	0.155	-0.088	0.011	1.41E-15	3613	0.108	-0.051	0.005	8.52E-29	17742
rs7412	19	50,103,919	t	c	0.071	-0.597	0.070	3.11E-17	3205	0.080	-0.045	0.005	2.26E-19	14455	0.080	-0.048	0.005	9.27E-22	17660
rs2075650	19	50,087,459	a	g	0.873	-0.027	0.004	1.65E-11	16068	0.863	-0.017	0.004	8.52E-06	21878	0.868	-0.022	0.003	3.22E-15	37946
rs4420638	19	50,114,786	a	g	0.841	-0.037	0.005	1.33E-12	13891	0.820	-0.017	0.004	3.31E-05	14600	0.828	-0.025	0.003	3.91E-15	28491
rs6857	19	50,084,094	t	c	0.132	0.029	0.004	4.79E-13	16067	0.140	0.014	0.004	3.12E-04	18477	0.136	0.022	0.003	1.75E-14	34544
rs4803763	19	50,049,131	c	g	0.283	0.021	0.003	1.92E-10	16696	0.237	0.026	0.009	5.49E-03	3613	0.279	0.021	0.003	5.65E-14	20309
rs1531517	19	49,934,013	a	g	0.065	-0.022	0.004	5.94E-07	18551	0.072	-0.023	0.005	7.30E-07	21939	0.068	-0.023	0.003	1.38E-13	40490
rs11083751	19	50,105,073	t	g	0.114	-0.503	0.071	1.42E-12	3205						0.114	-0.503	0.071	1.42E-12	3205
rs2927480	19	50,029,225	c	g	0.334	0.017	0.003	7.85E-10	16710	0.310	0.017	0.004	1.23E-04	7508	0.326	0.017	0.003	7.63E-12	24218
rs10402271	19	50,021,054	t	g	0.674	-0.016	0.003	1.95E-09	16714	0.686	-0.017	0.004	1.14E-04	7508	0.678	-0.016	0.003	4.63E-11	24222
rs7359852	19	50,027,875	t	c	0.667	-0.016	0.003	2.46E-09	16710	0.690	-0.017	0.004	1.19E-04	7508	0.674	-0.016	0.003	4.85E-11	24218
rs11751605	6	160,883,220	t	c	0.845	-0.019	0.004	5.82E-08	16732	0.832	-0.024	0.006	7.69E-05	7504	0.841	-0.020	0.003	7.82E-10	24236
rs646776	1	109,620,053	t	c	0.770	0.015	0.003	6.70E-07	16679	0.784	0.010	0.003	2.43E-04	21902	0.777	0.013	0.002	1.05E-09	38581
rs12740374	1	109,619,113	t	g	0.230	-0.015	0.003	9.41E-07	16677	0.216	-0.010	0.003	2.44E-04	21902	0.223	-0.013	0.002	1.05E-09	38579
rs2900478	12	21,260,064	a	t	0.165	0.016	0.003	2.26E-06	16731	0.164	0.017	0.006	3.54E-03	7504	0.165	0.016	0.003	1.22E-09	24235
rs2048327	6	160,783,522	t	c	0.626	-0.012	0.003	8.22E-07	16678	0.612	-0.018	0.004	1.64E-05	7507	0.621	-0.014	0.003	8.76E-09	24185
rs1985096	19	50,038,391	a	t	0.158	-0.022	0.004	2.60E-08	16253	0.167	-0.019	0.039	6.28E-01	212	0.158	-0.022	0.004	3.38E-08	16465
rs13166647	5	39,090,014	a	g	0.979	0.253	0.046	4.50E-08	2144						0.979	0.253	0.046	4.50E-08	2144
rs13172966	5	38,987,383	t	g	0.979	0.251	0.046	5.33E-08	2144						0.979	0.251	0.046	5.33E-08	2144
rs7696430	4	41,185,212	a	g	0.157	0.018	0.003	1.95E-07	16733	0.153	0.001	0.006	9.00E-01	7505	0.156	0.014	0.003	7.19E-08	24238
rs981844	4	154,871,478	a	g	0.747	0.011	0.002	9.36E-06	18151	0.751	0.001	0.005	8.27E-01	7508	0.748	0.010	0.002	2.13E-07	25659
rs11638450	15	78,977,587	t	c	0.365	-0.015	0.003	1.51E-05	16006	0.398	-0.008	0.004	6.51E-02	7501	0.376	-0.013	0.003	2.57E-07	23507
rs12428035	13	95,098,873	t	c	0.111	-0.015	0.003	9.52E-06	18576	0.109	-0.008	0.007	2.64E-01	7505	0.111	-0.014	0.003	5.13E-07	26081
rs12256987	10	15,555,973	a	t	0.982	-0.212	0.044	1.33E-06	4446						0.982	-0.212	0.044	1.33E-06	4446
rs9397844	6	156,178,867	t	c	0.932	0.030	0.006	1.41E-06	12963	0.934	0.005	0.016	7.71E-01	3613	0.932	0.027	0.006	1.76E-06	16576
rs17371021	4	154,883,745	a	g	0.246	-0.010	0.002	1.65E-05	18514	0.249	-0.001	0.005	8.72E-01	7491	0.246	-0.009	0.002	2.57E-06	26005
rs10026358	4	171,787,320	t	g	0.966	0.050	0.011	2.84E-06	6884						0.966	0.050	0.011	2.84E-06	6884
rs7072397	10	15,548,904	a	g	0.016	0.219	0.047	2.96E-06	4446						0.016	0.219	0.047	2.96E-06	4446
rs32506	5	55,608,212	t	c	0.027	-0.043	0.009	2.68E-06	10527	0.033	0.061	0.078	4.37E-01	212	0.027	-0.042	0.009	3.20E-06	10739

					First stage (n=18569)					Second stage (n=22318)					Combined (n=40887)				
SNPID	CHR	position	coded_all	noncoded_all	AF_coded_all	Effect*	SE	P-value	N	AF_coded_all	Effect*	SE	P-value	N	AF_coded_all	Effect*	SE	P-value	N
rs6478277	9	118,861,108	t	c	0.308	0.009	0.002	1.32E-04	8279	0.320	0.006	0.005	1.98E-01	7501	0.310	0.009	0.002	3.29E-06	15780
rs6739693	2	207,820,847	t	g	0.082	-0.018	0.004	2.38E-05	8392	0.080	-0.011	0.008	1.78E-01	7506	0.082	-0.017	0.004	3.51E-06	15898
rs12429385	13	95,099,937	a	g	0.889	0.014	0.003	1.44E-05	18564	0.893	0.004	0.012	7.52E-01	3613	0.889	0.013	0.003	4.03E-06	22177
rs4613264	2	230,651,695	a	g	0.589	0.010	0.002	2.88E-05	16679	0.581	0.001	0.004	8.35E-01	7503	0.588	0.008	0.002	4.12E-06	24182
rs28548376	4	171,806,901	t	g	0.027	-0.057	0.012	4.61E-06	6884						0.027	-0.057	0.012	4.61E-06	6884
rs11050607	12	30,077,778	a	g	0.981	-0.093	0.021	9.52E-06	8880	0.988	-0.063	0.058	2.79E-01	3613	0.982	-0.090	0.020	5.81E-06	12493
rs7334320	13	95,352,928	t	c	0.878	0.014	0.003	1.30E-05	18554	0.872	0.004	0.007	5.08E-01	7476	0.877	0.012	0.003	6.15E-06	26030
rs9516621	13	95,344,378	t	g	0.122	-0.014	0.003	1.19E-05	18557	0.121	-0.004	0.007	5.65E-01	7485	0.122	-0.012	0.003	7.26E-06	26042
rs10961118	9	13,535,346	a	g	0.017	-0.067	0.015	7.48E-06	3443						0.017	-0.067	0.015	7.48E-06	3443
rs1930096	9	110,393,856	a	g	0.092	-0.029	0.007	7.42E-05	6884	0.117	-0.022	0.013	8.91E-02	3401	0.098	-0.027	0.006	8.47E-06	10285
rs7835385	8	139,778,987	t	g	0.906	-0.019	0.005	6.10E-05	6558	0.904	-0.018	0.008	2.00E-02	7506	0.905	-0.019	0.004	8.48E-06	14064
rs2891833	2	220,471,528	t	c	0.237	0.014	0.003	3.48E-05	6558	0.243	0.004	0.005	4.50E-01	7481	0.239	0.011	0.003	1.10E-05	14039
rs1152493	14	55,870,761	c	g	0.119	-0.018	0.005	5.52E-05	6558	0.119	-0.017	0.007	1.21E-02	7502	0.119	-0.018	0.004	1.16E-05	14060
rs6687920	1	32,211,102	t	c	0.950	-0.029	0.007	8.32E-05	14793	0.939	-0.025	0.018	1.58E-01	3613	0.949	-0.029	0.007	1.23E-05	18406
rs17091112	14	55,732,898	a	c	0.933	0.024	0.006	3.94E-05	6558	0.948	0.018	0.010	6.96E-02	7506	0.937	0.022	0.005	1.31E-05	14064
rs2939334	5	39,577,794	c	g	0.046	-0.027	0.006	1.17E-05	16732	0.038	-0.009	0.011	4.18E-01	7494	0.044	-0.023	0.005	1.34E-05	24226
rs10145152	14	85,161,168	a	g	0.983	0.060	0.015	6.82E-05	9012	0.985	0.054	0.032	9.11E-02	3613	0.983	0.059	0.014	1.43E-05	12625
rs17756944	11	78,923,977	t	c	0.051	-0.031	0.007	7.48E-06	6541	0.030	-0.001	0.030	9.71E-01	3613	0.050	-0.029	0.007	1.56E-05	10154
rs4149000	12	21,339,264	t	c	0.125	0.017	0.004	1.53E-05	14871	0.133	0.009	0.007	1.85E-01	7497	0.127	0.015	0.003	1.60E-05	22368
rs3797135	5	10,782,757	c	g	0.072	-0.025	0.005	6.08E-06	6558	0.073	0.017	0.014	2.21E-01	3613	0.072	-0.020	0.005	1.65E-05	10171
rs7586037	2	11,530,421	a	g	0.014	-0.052	0.012	2.11E-05	15897	0.016	0.012	0.106	9.08E-01	212	0.014	-0.051	0.012	1.76E-05	16109
rs11144515	9	77,416,534	a	c	0.080	0.017	0.004	7.35E-05	8403	0.075	0.035	0.054	5.17E-01	212	0.080	0.017	0.004	1.82E-05	8615
rs4142173	2	220,460,776	t	c	0.258	0.013	0.003	3.34E-05	6558	0.248	0.005	0.005	3.05E-01	7481	0.255	0.011	0.003	2.20E-05	14039
rs1399708	3	136,037,577	t	c	0.140	0.012	0.003	1.21E-04	18575	0.141	0.008	0.006	1.74E-01	7507	0.140	0.011	0.003	2.88E-05	26082
rs1080022	6	144,706,424	t	c	0.954	-0.024	0.006	1.53E-04	16731	0.951	-0.022	0.018	2.31E-01	3613	0.954	-0.024	0.006	2.99E-05	20344
rs6980143	7	100,421,196	c	g	0.413	0.017	0.004	1.44E-05	5327	0.437	-0.006	0.025	8.24E-01	212	0.414	0.016	0.004	3.14E-05	5539
rs8055160	16	12,844,157	a	g	0.019	0.102	0.024	3.37E-05	5135						0.019	0.102	0.024	3.37E-05	5135
rs6494593	15	30,771,488	a	g	0.017	-0.069	0.017	3.40E-05	6884						0.017	-0.069	0.017	3.40E-05	6884
rs11708473	3	36,496,268	t	c	0.285	-0.013	0.003	3.45E-05	14793	0.283	-0.001	0.009	9.06E-01	3613	0.285	-0.012	0.003	3.54E-05	18406
rs17236593	3	161,927,549	c	g	0.838	-0.016	0.004	3.20E-05	16610	0.822	-0.012	0.012	3.05E-01	3613	0.836	-0.016	0.004	3.81E-05	20223
rs17343443	10	42,506,141	a	g	0.920	-0.020	0.005	3.72E-05	16452	0.919	-0.015	0.015	3.15E-01	3613	0.920	-0.020	0.005	3.92E-05	20065
rs16848588	3	185,982,039	a	c	0.017	-0.097	0.024	4.32E-05	10617	0.012	-0.289	0.303	3.40E-01	212	0.017	-0.098	0.024	4.05E-05	10829

					First stage (n=18569)					Second stage (n=22318)					Combined (n=40887)				
SNPID	CHR	position	coded_all	noncoded_all	AF_coded_all	Effect*	SE	P-value	N	AF_coded_all	Effect*	SE	P-value	N	AF_coded_all	Effect*	SE	P-value	N
rs10994219	10	61,572,095	t	c	0.020	0.110	0.024	5.90E-06	3445	0.025	0.009	0.042	8.37E-01	3613	0.021	0.085	0.021	4.50E-05	7058
rs9459520	6	166,219,798	a	c	0.926	0.041	0.010	7.30E-05	12615	0.923	-0.003	0.089	9.76E-01	212	0.926	0.041	0.010	4.69E-05	12827
rs6884324	5	62,213,017	t	c	0.092	-0.026	0.006	3.18E-06	6524	0.109	-0.004	0.013	7.44E-01	3613	0.095	-0.022	0.005	4.72E-05	10137
rs11896096	2	11,491,548	t	c	0.985	0.049	0.012	3.39E-05	16207	0.984	-0.018	0.103	8.64E-01	212	0.985	0.048	0.012	5.43E-05	16419
rs4710104	6	167,133,924	a	g	0.984	0.044	0.011	8.77E-05	12456	0.981	0.023	0.028	4.15E-01	3613	0.984	0.041	0.010	5.82E-05	16069
rs909078	3	121,846,997	a	t	0.728	0.029	0.007	6.25E-05	4727						0.728	0.029	0.007	6.25E-05	4727
rs10484520	6	167,134,676	a	g	0.017	-0.044	0.011	7.01E-05	12456	0.020	-0.021	0.027	4.31E-01	3613	0.017	-0.041	0.010	6.31E-05	16069
rs4301085	4	150,573,294	a	g	0.970	-0.057	0.014	5.19E-05	9353	0.962	-0.021	0.030	4.80E-01	3613	0.969	-0.051	0.013	6.65E-05	12966
rs4510455	4	150,570,517	a	g	0.970	-0.057	0.014	6.04E-05	9351	0.962	-0.021	0.030	4.80E-01	3613	0.969	-0.051	0.013	6.65E-05	12964
rs1244983	1	213,098,237	a	g	0.545	0.011	0.003	1.02E-05	16671	0.542	0.012	0.008	1.15E-01	3613	0.545	0.011	0.003	6.66E-05	20284
rs4858940	3	88,337,510	t	c	0.111	-0.014	0.003	1.44E-05	18576	0.112	0.005	0.007	4.89E-01	7506	0.111	-0.011	0.003	6.95E-05	26082
rs17452465	2	115,464,189	a	t	0.983	0.049	0.012	3.39E-05	10527	0.978	-0.100	0.110	3.67E-01	212	0.983	0.047	0.012	7.44E-05	10739
rs1112364	17	57,597,131	t	c	0.095	0.017	0.004	1.24E-04	16732	0.102	0.003	0.007	6.69E-01	7504	0.097	0.014	0.004	7.94E-05	24236
rs1837760	4	60,705,624	a	t	0.066	0.018	0.004	3.11E-05	18553	0.070	-0.002	0.008	7.88E-01	7500	0.067	0.014	0.004	8.02E-05	26053
rs10190944	2	129,478,715	a	g	0.894	0.027	0.007	9.47E-05	6884						0.894	0.027	0.007	9.47E-05	6884
rs10768434	11	4,962,876	t	g	0.389	-0.011	0.003	1.18E-04	6504	0.400	-0.007	0.004	1.19E-01	7502	0.393	-0.010	0.003	9.67E-05	14006
rs1470175	4	60,694,605	a	t	0.934	-0.018	0.004	3.45E-05	18544	0.930	0.003	0.008	7.00E-01	7455	0.933	-0.014	0.004	9.87E-05	25999
rs1333472	13	46,662,596	a	t	0.804	0.016	0.003	6.70E-07	16729	0.789	-0.010	0.006	7.76E-02	7394	0.801	0.010	0.003	1.05E-04	24123
rs11672562	19	61,020,253	a	g	0.114	-0.014	0.003	4.20E-05	18568	0.111	0.007	0.007	2.93E-01	7504	0.114	-0.011	0.003	1.31E-04	26072
rs9359584	6	85,094,893	t	c	0.515	0.018	0.004	4.73E-06	5330	0.501	0.003	0.004	4.20E-01	7508	0.508	0.011	0.003	1.36E-04	12838
rs9353173	6	85,095,268	a	g	0.516	0.018	0.004	5.35E-06	5330	0.501	0.003	0.004	4.21E-01	7508	0.509	0.011	0.003	1.37E-04	12838
rs16882272	5	53,459,882	t	c	0.021	0.083	0.020	2.54E-05	3445	0.016	0.007	0.042	8.74E-01	3613	0.020	0.069	0.018	1.37E-04	7058
rs2824332	21	17,785,838	t	c	0.221	0.014	0.003	4.03E-05	6558	0.219	-0.002	0.005	6.84E-01	7462	0.220	0.010	0.003	1.39E-04	14020
rs2492739	10	87,988,946	c	g	0.705	0.018	0.005	1.00E-04	6881	0.673	0.012	0.009	1.74E-01	3401	0.697	0.017	0.004	1.43E-04	10282
rs10267283	7	30,377,310	c	g	0.984	0.059	0.015	5.89E-05	13446	0.980	0.020	0.029	4.91E-01	3613	0.983	0.051	0.013	1.43E-04	17059
rs11149063	13	76,734,134	t	c	0.938	0.022	0.005	4.00E-05	16260	0.939	-0.001	0.009	9.15E-01	7421	0.938	0.017	0.004	1.45E-04	23681
rs6994586	8	117,776,909	c	g	0.052	0.036	0.008	9.98E-06	12963	0.070	-0.030	0.023	1.85E-01	3613	0.054	0.029	0.008	1.45E-04	16576
rs17817345	18	70,370,317	a	g	0.259	0.012	0.003	1.33E-04	14481	0.266	0.004	0.005	4.73E-01	7502	0.261	0.010	0.003	1.46E-04	21983
rs7085784	10	70,947,335	c	g	0.053	-0.028	0.007	8.89E-05	4619	0.056	-0.004	0.018	8.27E-01	3613	0.054	-0.025	0.007	1.47E-04	8232
rs4986850	17	38,498,997	t	c	0.085	-0.020	0.005	2.88E-05	16730	0.076	0.001	0.015	9.74E-01	3613	0.084	-0.018	0.005	1.56E-04	20343
rs1571755	1	230,381,128	t	c	0.371	-0.008	0.002	3.01E-04	18514	0.373	-0.001	0.004	7.51E-01	7489	0.371	-0.007	0.002	1.69E-04	26003
rs4245188	11	110,802,759	a	g	0.965	0.029	0.007	5.55E-05	14605	0.960	0.002	0.014	8.90E-01	4106	0.964	0.023	0.006	1.72E-04	18711

					First stage (n=18569)					Second stage (n=22318)					Combined (n=40887)				
SNPID	CHR	position	coded_all	noncoded_all	AF_coded_all	Effect*	SE	P-value	N	AF_coded_all	Effect*	SE	P-value	N	AF_coded_all	Effect*	SE	P-value	N
rs495331	18	18,333,321	a	g	0.739	0.011	0.003	1.26E-04	16677	0.789	0.006	0.005	2.42E-01	7504	0.752	0.010	0.003	1.77E-04	24181
rs480159	18	18,336,828	t	g	0.739	0.011	0.003	1.26E-04	16679	0.777	0.006	0.005	2.37E-01	7504	0.749	0.010	0.003	1.78E-04	24183
rs11234750	11	86,105,386	t	c	0.039	-0.031	0.008	6.64E-05	12962	0.027	-0.002	0.029	9.57E-01	3613	0.038	-0.029	0.008	1.80E-04	16575
rs2478553	9	77,536,890	a	g	0.972	-0.032	0.009	2.24E-04	12963	0.957	-0.025	0.022	2.46E-01	3613	0.970	-0.031	0.008	1.92E-04	16576
rs6537666	1	110,884,044	a	g	0.690	-0.012	0.003	1.18E-05	16679	0.701	-0.003	0.005	5.38E-01	7506	0.693	-0.010	0.003	1.92E-04	24185
rs11102127	1	110,891,290	t	g	0.696	-0.012	0.003	5.95E-06	16677	0.701	-0.003	0.005	5.42E-01	7504	0.697	-0.010	0.003	1.93E-04	24181
rs4689568	4	7,012,490	t	c	0.825	-0.012	0.003	1.17E-04	16733	0.822	0.004	0.010	7.15E-01	3613	0.825	-0.011	0.003	2.02E-04	20346
rs17813281	12	68,002,012	a	g	0.125	-0.019	0.005	3.29E-05	12961	0.122	-0.003	0.019	8.89E-01	3613	0.125	-0.018	0.005	2.11E-04	16574
rs7576451	2	230,628,830	a	g	0.271	-0.011	0.003	8.13E-05	16732	0.288	-0.005	0.005	2.80E-01	7503	0.276	-0.010	0.003	2.19E-04	24235
rs9876212	3	71,578,164	a	g	0.187	-0.012	0.003	9.22E-06	18569	0.198	-0.002	0.005	6.38E-01	7508	0.190	-0.010	0.003	2.21E-04	26077
rs9422671	10	42,608,632	t	g	0.926	0.018	0.004	2.80E-05	15112	0.922	-0.005	0.008	4.93E-01	7503	0.925	0.013	0.004	2.31E-04	22615
rs7865666	9	4,368,591	c	g	0.659	0.012	0.003	1.30E-04	6502	0.654	-0.002	0.008	7.95E-01	3613	0.658	0.010	0.003	2.35E-04	10115
rs7754232	6	134,779,102	a	g	0.676	0.013	0.003	5.43E-05	12964	0.690	-0.011	0.009	2.08E-01	3613	0.678	0.010	0.003	2.40E-04	16577
rs8058775	16	71,612,295	t	c	0.986	0.044	0.012	2.48E-04	12684						0.986	0.044	0.012	2.48E-04	12684
rs1158096	8	13,415,412	t	c	0.969	0.032	0.008	1.05E-04	14460	0.971	0.006	0.013	6.56E-01	7508	0.970	0.025	0.007	2.56E-04	21968
rs12586805	14	60,650,202	t	c	0.981	-0.063	0.015	4.52E-05	11511	0.965	-0.008	0.021	6.96E-01	3613	0.976	-0.045	0.012	2.61E-04	15124
rs1229351	1	165,909,616	t	c	0.263	0.014	0.003	3.68E-05	10527	0.260	-0.007	0.006	2.49E-01	4105	0.262	0.010	0.003	2.75E-04	14632
rs17159167	5	106,213,288	a	g	0.934	0.022	0.005	5.10E-05	16719	0.928	-0.023	0.015	1.07E-01	3613	0.933	0.017	0.005	2.82E-04	20332
rs17049422	2	58,307,564	t	c	0.033	0.023	0.006	1.67E-04	18283	0.030	-0.008	0.024	7.37E-01	3613	0.033	0.021	0.006	2.83E-04	21896
rs11619378	13	107,679,090	t	g	0.878	0.015	0.004	2.41E-04	6557	0.878	0.003	0.012	8.09E-01	3613	0.878	0.014	0.004	2.90E-04	10170
rs3742103	13	76,638,710	t	c	0.936	0.021	0.005	5.95E-05	16732	0.939	-0.001	0.009	8.89E-01	7421	0.937	0.016	0.004	3.08E-04	24153
rs17470697	18	24,171,538	a	g	0.973	0.038	0.010	2.26E-04	4619	0.970	0.006	0.024	7.85E-01	3613	0.973	0.033	0.009	3.13E-04	8232
rs2572324	4	90,897,821	a	g	0.691	0.012	0.003	1.14E-04	6557	0.707	0.002	0.005	7.39E-01	7480	0.695	0.009	0.003	3.31E-04	14037
rs7706073	5	106,236,875	a	g	0.067	-0.022	0.005	3.65E-05	16728	0.074	0.024	0.014	8.84E-02	3613	0.068	-0.017	0.005	3.34E-04	20341
rs13418724	2	58,304,037	a	g	0.034	0.023	0.006	1.57E-04	18285	0.029	-0.012	0.024	6.00E-01	3613	0.034	0.021	0.006	3.37E-04	21898
rs9439755	1	29,886,376	t	c	0.729	0.012	0.003	1.55E-04	16731	0.769	0.001	0.005	7.80E-01	7506	0.739	0.009	0.003	3.43E-04	24237
rs181489	4	90,854,043	t	c	0.305	-0.012	0.003	8.71E-05	6501	0.293	-0.001	0.005	7.73E-01	7480	0.302	-0.009	0.003	3.48E-04	13981
rs13148401	4	40,754,704	t	c	0.688	-0.012	0.003	6.45E-06	16675	0.699	-0.002	0.005	7.45E-01	7490	0.691	-0.009	0.003	3.52E-04	24165
rs812334	3	77,783,760	a	g	0.512	-0.011	0.003	4.15E-05	6503	0.547	-0.004	0.004	3.16E-01	7508	0.524	-0.009	0.002	3.64E-04	14011
rs17247149	17	68,135,016	a	g	0.983	0.042	0.011	1.62E-04	4619	0.975	-0.115	0.076	1.31E-01	212	0.983	0.039	0.011	3.67E-04	4831
rs1163750	3	77,781,132	a	g	0.532	-0.011	0.003	1.02E-04	6504	0.566	-0.004	0.004	3.28E-01	7508	0.543	-0.009	0.002	3.82E-04	14012
rs7018761	9	71,752,033	a	g	0.435	0.011	0.003	1.04E-04	6504	0.428	0.002	0.008	7.62E-01	3613	0.434	0.010	0.003	4.09E-04	10117

					First stage (n=18569)					Second stage (n=22318)					Combined (n=40887)				
SNPID	CHR	position	coded_all	noncoded_all	AF_coded_all	Effect*	SE	P-value	N	AF_coded_all	Effect*	SE	P-value	N	AF_coded_all	Effect*	SE	P-value	N
rs13279426	8	3,927,889	a	g	0.115	0.017	0.004	2.40E-05	16707	0.100	-0.007	0.009	4.16E-01	4045	0.113	0.013	0.004	4.16E-04	20752
rs6595305	5	120,933,624	t	c	0.672	-0.012	0.003	1.95E-05	16718	0.663	0.006	0.008	4.78E-01	3613	0.671	-0.010	0.003	4.16E-04	20331
rs988449	20	10,657,411	t	c	0.020	0.138	0.041	7.31E-04	6138	0.045	0.061	0.034	7.41E-02	3613	0.035	0.092	0.026	4.27E-04	9751
rs9516774	13	96,316,071	a	c	0.460	-0.009	0.002	5.74E-05	18518	0.459	0.006	0.004	1.96E-01	7506	0.460	-0.006	0.002	4.43E-04	26024
rs10086299	8	11,522,668	a	g	0.923	0.020	0.005	8.99E-05	14876	0.923	-0.014	0.015	3.37E-01	3613	0.923	0.017	0.005	5.08E-04	18489
rs3763547	8	52,974,896	a	g	0.084	-0.020	0.005	2.13E-05	16258	0.101	-0.002	0.007	7.80E-01	7495	0.089	-0.014	0.004	5.16E-04	23753
rs2111326	10	101,352,526	a	g	0.504	-0.010	0.003	3.55E-05	16673	0.500	-0.007	0.008	3.56E-01	3613	0.503	-0.010	0.003	5.82E-04	20286
rs10018539	4	181,824,985	t	g	0.986	-0.113	0.033	5.83E-04	3370						0.986	-0.113	0.033	5.83E-04	3370
rs1481736	8	32,100,184	a	g	0.429	-0.013	0.003	9.20E-06	6503	0.429	0.001	0.004	8.77E-01	7507	0.429	-0.008	0.002	5.84E-04	14010
rs4412196	6	11,945,258	a	c	0.184	0.010	0.003	3.09E-04	18571	0.171	0.006	0.006	3.26E-01	7505	0.181	0.009	0.003	6.51E-04	26076
rs2327487	6	11,943,141	a	t	0.816	-0.010	0.003	2.68E-04	18573	0.829	-0.006	0.006	3.27E-01	7505	0.819	-0.009	0.003	6.51E-04	26078
rs1524996	4	114,505,285	t	c	0.850	-0.017	0.004	5.22E-06	14792	0.845	0.018	0.011	9.86E-02	3613	0.849	-0.013	0.004	6.58E-04	18405
rs7655563	4	7,044,838	a	g	0.174	0.012	0.003	2.38E-04	16731	0.176	-0.002	0.006	7.69E-01	7505	0.175	0.009	0.003	6.85E-04	24236
rs10866563	5	4,303,214	a	g	0.190	-0.015	0.004	6.66E-05	6558	0.187	-0.003	0.006	6.15E-01	7495	0.189	-0.011	0.003	6.95E-04	14053
rs10979269	9	109,995,229	t	c	0.896	0.023	0.006	4.59E-05	4619	0.898	-0.005	0.014	7.29E-01	3613	0.896	0.019	0.006	7.18E-04	8232
rs150344	16	22,728,560	a	c	0.238	0.012	0.003	4.53E-05	16677	0.240	-0.001	0.005	9.18E-01	7507	0.239	0.009	0.003	7.23E-04	24184
rs219608	16	61,918,107	c	g	0.221	0.012	0.003	1.55E-04	16730	0.201	-0.001	0.005	8.86E-01	7506	0.216	0.009	0.003	7.24E-04	24236
rs758491	16	7,621,779	t	c	0.661	0.011	0.003	1.73E-04	6504	0.668	0.002	0.005	6.27E-01	7502	0.663	0.009	0.003	7.41E-04	14006
rs6994000	8	13,394,011	t	g	0.032	-0.029	0.008	1.93E-04	16454	0.030	-0.007	0.013	6.02E-01	7508	0.031	-0.023	0.007	7.46E-04	23962
rs715660	3	36,478,071	a	c	0.647	0.011	0.003	3.65E-05	16733	0.650	0.003	0.004	5.21E-01	7503	0.648	0.008	0.003	7.55E-04	24236
rs4655544	1	67,718,990	t	c	0.029	-0.034	0.009	1.67E-04	6558	0.040	0.013	0.024	5.83E-01	3613	0.030	-0.028	0.008	7.98E-04	10171
rs10134441	14	47,866,273	c	g	0.127	-0.018	0.005	1.73E-04	4619	0.152	-0.001	0.012	9.50E-01	3613	0.131	-0.015	0.005	8.32E-04	8232
rs12621900	2	207,826,546	t	c	0.227	-0.012	0.003	7.88E-06	8388	0.227	0.014	0.009	1.33E-01	3613	0.227	-0.010	0.003	8.51E-04	12001
rs11193300	10	109,026,100	a	g	0.867	0.015	0.004	1.98E-04	16687	0.877	0.002	0.007	8.10E-01	7508	0.870	0.011	0.003	8.59E-04	24195
rs855356	6	9,936,690	c	g	0.637	0.011	0.003	1.56E-05	16670	0.649	-0.003	0.008	7.18E-01	3613	0.638	0.009	0.003	9.07E-04	20283
rs657320	11	125,924,111	a	g	0.127	-0.020	0.005	1.92E-04	6558	0.130	0.001	0.008	9.25E-01	4102	0.128	-0.014	0.004	9.15E-04	10660
rs723318	6	107,387,007	t	c	0.601	0.011	0.003	1.56E-04	6482	0.598	-0.003	0.008	7.36E-01	3613	0.601	0.009	0.003	9.35E-04	10095
rs9316263	13	46,664,581	t	c	0.770	0.014	0.003	1.83E-06	16733	0.785	-0.010	0.006	8.88E-02	7394	0.773	0.009	0.003	9.38E-04	24127
rs17443266	12	19,060,720	a	g	0.019	-0.035	0.010	5.42E-04	16452	0.019	0.006	0.033	8.64E-01	3613	0.019	-0.032	0.010	9.73E-04	20065
rs9527895	13	58,265,768	t	c	0.823	-0.016	0.004	1.37E-05	6555	0.830	0.000	0.006	9.96E-01	7506	0.825	-0.011	0.003	9.83E-04	14061
rs12761509	10	109,062,446	t	g	0.123	-0.015	0.004	1.32E-04	14835	0.121	-0.001	0.007	8.79E-01	7508	0.123	-0.011	0.003	1.00E-03	22343
rs17448081	18	24,135,055	t	g	0.031	-0.036	0.010	1.79E-04	4619	0.037	0.000	0.022	9.84E-01	3613	0.032	-0.030	0.009	1.01E-03	8232

SNPID	CHR	position	coded_all	noncoded_all	First stage (n=18569)					Second stage (n=22318)					Combined (n=40887)				
					AF_coded_all	Effect*	SE	P-value	N	AF_coded_all	Effect*	SE	P-value	N	AF_coded_all	Effect*	SE	P-value	N
rs7575586	2	180,079,084	t	c	0.226	-0.012	0.003	5.30E-05	16731	0.222	0.002	0.005	7.36E-01	7458	0.225	-0.008	0.003	1.11E-03	24189
rs10220469	14	60,659,081	a	c	0.012	0.067	0.019	4.56E-04	10617	0.021	-0.074	0.086	3.88E-01	212	0.012	0.060	0.019	1.13E-03	10829
rs900662	2	52,709,806	a	g	0.960	0.034	0.010	5.95E-04	12211	0.981	-0.126	0.099	2.02E-01	212	0.960	0.032	0.010	1.14E-03	12423
rs4891322	18	63,408,553	t	c	0.786	-0.012	0.003	1.56E-05	8389	0.787	0.002	0.005	7.18E-01	7507	0.786	-0.008	0.003	1.14E-03	15896
rs1710884	3	10,994,209	a	g	0.268	-0.015	0.004	3.89E-05	12962	0.247	0.007	0.010	5.07E-01	3613	0.265	-0.012	0.004	1.15E-03	16575
rs730258	4	68,431,265	a	g	0.819	0.015	0.004	8.34E-05	6558	0.820	0.002	0.006	7.86E-01	7469	0.819	0.011	0.003	1.17E-03	14027
rs17756848	11	78,913,670	a	g	0.935	0.024	0.006	3.69E-05	6558	0.930	-0.004	0.010	7.23E-01	4087	0.934	0.017	0.005	1.22E-03	10645
rs2222174	2	180,094,031	t	c	0.774	0.012	0.003	8.05E-05	16732	0.779	-0.002	0.005	6.91E-01	7496	0.775	0.008	0.003	1.23E-03	24228
rs17004546	21	43,664,566	a	g	0.046	-0.036	0.010	3.17E-04	12963	0.040	0.001	0.021	9.77E-01	3613	0.045	-0.029	0.009	1.24E-03	16576
rs3935586	3	187,657,324	t	c	0.316	-0.011	0.003	9.53E-05	16679	0.312	-0.001	0.005	8.38E-01	7506	0.315	-0.008	0.003	1.25E-03	24185
rs4673074	2	224,304,377	a	t	0.502	-0.010	0.003	8.23E-05	16678	0.491	-0.004	0.004	3.81E-01	7485	0.498	-0.008	0.002	1.26E-03	24163
rs1875196	5	1,744,202	c	g	0.988	0.067	0.016	2.96E-05	2069	0.983	-0.041	0.033	2.13E-01	3401	0.987	0.046	0.014	1.27E-03	5470
rs10789233	1	67,717,772	a	g	0.970	0.034	0.009	1.27E-04	6558	0.972	-0.021	0.023	3.70E-01	3613	0.970	0.027	0.008	1.37E-03	10171
rs17247213	17	68,140,355	a	g	0.018	-0.038	0.011	3.85E-04	6556	0.025	0.134	0.079	9.00E-02	212	0.018	-0.035	0.011	1.44E-03	6768
rs17502589	12	19,058,244	c	g	0.981	0.037	0.011	4.61E-04	14514	0.981	-0.003	0.033	9.18E-01	3613	0.981	0.033	0.010	1.59E-03	18127
rs3107261	6	107,399,255	t	c	0.410	-0.011	0.003	8.87E-05	6488	0.415	0.007	0.008	3.99E-01	3613	0.411	-0.009	0.003	1.60E-03	10101
rs17069047	8	3,966,391	t	c	0.100	0.017	0.004	7.01E-05	16691	0.100	-0.009	0.007	2.12E-01	7446	0.100	0.011	0.004	1.64E-03	24137
rs3822414	5	10,755,688	t	c	0.063	-0.027	0.006	2.47E-06	6558	0.061	0.009	0.009	2.98E-01	7506	0.062	-0.016	0.005	1.64E-03	14064
rs11666496	19	61,021,740	a	c	0.889	0.017	0.004	1.31E-05	16733	0.889	-0.007	0.007	3.32E-01	7504	0.889	0.011	0.003	1.66E-03	24237
rs7716942	5	120,841,745	a	t	0.294	0.011	0.003	8.18E-05	16730	0.293	0.000	0.005	9.85E-01	7505	0.294	0.008	0.003	1.71E-03	24235
rs7720025	5	153,923,439	t	c	0.373	-0.011	0.003	5.07E-05	16677	0.372	-0.001	0.004	8.47E-01	7506	0.373	-0.008	0.003	1.89E-03	24183
rs4429899	5	153,912,635	a	t	0.627	0.011	0.003	5.07E-05	16677	0.628	0.001	0.004	8.48E-01	7506	0.627	0.008	0.003	1.89E-03	24183
rs13267697	8	70,275,487	t	g	0.914	0.020	0.005	7.01E-05	16650	0.915	-0.032	0.015	2.91E-02	3613	0.914	0.015	0.005	2.05E-03	20263
rs2217533	1	238,114,921	a	g	0.887	-0.014	0.004	7.48E-05	8394	0.892	-0.001	0.007	9.01E-01	7505	0.888	-0.011	0.003	2.13E-03	15899
rs1933463	6	97,624,724	a	g	0.189	0.016	0.004	6.30E-05	15559	0.197	-0.003	0.006	6.59E-01	7496	0.192	0.010	0.003	2.49E-03	23055
rs1444115	18	63,395,621	t	c	0.782	-0.015	0.004	1.70E-05	6553	0.779	-0.001	0.005	8.87E-01	7506	0.781	-0.010	0.003	2.49E-03	14059
rs6839279	4	68,472,373	t	g	0.154	-0.015	0.004	8.25E-05	6558	0.159	0.001	0.006	8.70E-01	7507	0.156	-0.010	0.003	2.65E-03	14065
rs646336	6	56,378,593	a	g	0.775	0.011	0.003	1.59E-04	16726	0.778	-0.001	0.005	7.76E-01	7505	0.776	0.008	0.003	2.68E-03	24231
rs840708	11	4,908,175	t	c	0.550	-0.011	0.003	2.05E-04	6499	0.522	0.000	0.004	9.82E-01	7508	0.541	-0.007	0.002	2.70E-03	14007
rs6696040	1	70,442,900	t	c	0.920	0.018	0.005	2.82E-04	14794	0.922	-0.001	0.008	8.84E-01	7508	0.921	0.013	0.004	2.71E-03	22302
rs9538189	13	58,231,777	c	g	0.825	-0.014	0.004	1.06E-04	6550	0.832	-0.001	0.006	8.71E-01	7506	0.827	-0.010	0.003	2.82E-03	14056
rs12547959	8	32,101,314	a	g	0.428	-0.013	0.003	1.48E-05	6503	0.443	0.004	0.004	3.18E-01	7493	0.433	-0.007	0.002	3.18E-03	13996

SNPID	CHR	position	coded_all	noncoded_all	First stage (n=18569) AF_coded_all	Effect*	SE	P-value	N	Second stage (n=22318) AF_coded_all	Effect*	SE	P-value	N	Combined (n=40887) AF_coded_all	Effect*	SE	P-value	N
rs2980569	8	110,416,578	a	c	0.536	0.011	0.003	8.87E-05	6491	0.530	-0.011	0.008	1.54E-01	3613	0.535	0.008	0.003	3.42E-03	10104
rs571592	1	242,171,257	t	c	0.361	-0.011	0.003	3.15E-04	6504	0.348	0.002	0.005	6.55E-01	7494	0.357	-0.007	0.003	3.80E-03	13998
rs280349	6	50,629,885	a	c	0.962	0.028	0.007	9.90E-05	14623	0.968	-0.016	0.013	2.18E-01	7504	0.963	0.018	0.006	3.81E-03	22127
rs4238534	15	80,351,610	t	c	0.262	0.011	0.003	2.07E-05	8344	0.260	-0.002	0.005	6.26E-01	7507	0.262	0.007	0.003	3.88E-03	15851
rs9953989	18	57,174,729	t	c	0.946	0.025	0.006	7.50E-05	14612	0.940	-0.010	0.009	2.69E-01	7507	0.944	0.014	0.005	4.10E-03	22119
rs4859003	3	88,330,335	t	c	0.103	-0.015	0.004	1.39E-05	18544	0.111	0.005	0.007	4.67E-01	7506	0.105	-0.010	0.003	4.38E-03	26050
rs2340988	16	23,891,757	a	c	0.535	-0.010	0.003	3.55E-05	16679	0.531	-0.001	0.004	8.20E-01	7507	0.534	-0.007	0.002	4.43E-03	24186
rs1889180	13	23,013,747	t	c	0.407	0.008	0.002	3.21E-04	8345	0.409	-0.011	0.005	2.13E-02	4075	0.407	0.005	0.002	4.61E-03	12420
rs2293324	4	114,513,757	t	c	0.853	-0.015	0.004	3.82E-05	16732	0.874	0.004	0.006	4.90E-01	7507	0.859	-0.010	0.003	4.85E-03	24239
rs7956312	12	26,765,783	a	t	0.977	-0.040	0.010	5.60E-05	6558	0.980	0.012	0.014	4.01E-01	7505	0.978	-0.023	0.008	5.02E-03	14063
rs722661	7	13,878,170	a	c	0.411	-0.011	0.003	1.76E-05	16678	0.416	0.001	0.004	7.50E-01	7506	0.413	-0.007	0.002	5.08E-03	24184
rs9847558	3	108,471,024	c	g	0.719	0.012	0.003	3.36E-05	16676	0.739	-0.006	0.005	2.21E-01	7456	0.724	0.007	0.003	5.31E-03	24132
rs9965819	18	57,175,532	t	c	0.067	-0.024	0.006	4.17E-05	14611	0.060	0.010	0.009	2.69E-01	7507	0.065	-0.014	0.005	6.33E-03	22118
rs228914	22	35,834,458	a	c	0.969	0.074	0.019	8.95E-05	5135	0.873	0.008	0.013	5.18E-01	3613	0.903	0.029	0.011	6.45E-03	8748
rs382758	13	112,448,170	a	g	0.145	-0.014	0.004	1.11E-04	16725	0.156	0.002	0.006	7.43E-01	7505	0.148	-0.009	0.003	6.53E-03	24230
rs700940	2	6,878,379	t	c	0.531	-0.010	0.003	2.43E-04	16206	0.512	0.000	0.004	9.93E-01	7496	0.525	-0.007	0.002	6.61E-03	23702
rs2170639	12	26,775,647	a	g	0.976	-0.038	0.010	7.91E-05	6558	0.980	0.011	0.014	4.63E-01	7505	0.977	-0.022	0.008	6.92E-03	14063
rs4889954	17	75,680,189	a	g	0.695	-0.010	0.003	1.73E-04	16679	0.690	0.001	0.005	7.77E-01	7506	0.694	-0.007	0.003	7.17E-03	24185
rs17043126	2	52,707,417	t	c	0.029	-0.043	0.012	2.53E-04	10527	0.055	0.022	0.022	3.14E-01	3613	0.035	-0.028	0.011	7.42E-03	14140
rs17240471	15	88,172,175	a	g	0.491	-0.011	0.003	2.10E-05	16675	0.502	0.002	0.004	5.91E-01	7407	0.495	-0.007	0.002	7.54E-03	24082
rs13281179	8	52,909,305	c	g	0.055	-0.022	0.006	1.97E-04	16254	0.055	0.009	0.010	3.78E-01	7448	0.055	-0.014	0.005	7.55E-03	23702
rs1338172	10	120,353,701	a	g	0.881	0.014	0.004	1.07E-04	8397	0.881	-0.004	0.007	5.10E-01	7507	0.881	0.009	0.003	7.77E-03	15904
rs9824973	3	108,527,984	t	c	0.698	0.011	0.003	7.01E-05	16676	0.711	-0.005	0.005	3.23E-01	7493	0.702	0.007	0.003	8.80E-03	24169
rs17412858	13	109,050,609	a	g	0.628	0.010	0.003	2.81E-04	16676	0.629	-0.001	0.004	8.56E-01	7496	0.628	0.006	0.002	8.96E-03	24172
rs10516149	5	178,386,724	t	g	0.964	-0.032	0.008	2.10E-05	6558	0.963	0.016	0.012	1.96E-01	7497	0.964	-0.017	0.007	9.17E-03	14055
rs943810	10	130,245,170	t	c	0.406	-0.011	0.003	1.79E-04	6504	0.417	0.003	0.004	5.08E-01	7494	0.410	-0.006	0.002	9.24E-03	13998
rs11219884	11	98,808,987	c	g	0.047	0.042	0.010	5.04E-05	10054	0.207	-0.006	0.010	5.77E-01	3613	0.126	0.018	0.007	9.53E-03	13667
rs9296849	6	56,482,713	a	c	0.706	0.010	0.003	2.56E-04	16729	0.694	-0.002	0.005	6.25E-01	7504	0.703	0.007	0.003	9.87E-03	24233
rs9515085	13	109,056,783	t	g	0.603	0.010	0.003	1.15E-04	16652	0.625	-0.001	0.004	8.10E-01	7496	0.610	0.006	0.002	9.88E-03	24148
rs13067689	3	13,795,614	a	g	0.514	-0.011	0.003	5.97E-05	16677	0.510	0.003	0.004	4.78E-01	7500	0.513	-0.006	0.002	1.01E-02	24177
rs11957895	5	148,196,885	a	g	0.277	-0.097	0.023	3.44E-05	2075	0.171	-0.002	0.017	9.05E-01	3401	0.208	-0.035	0.014	1.01E-02	5476
rs8123349	20	23,584,830	a	g	0.876	0.016	0.004	8.63E-05	16732	0.866	-0.012	0.007	8.90E-02	7493	0.873	0.009	0.003	1.01E-02	24225

					First stage (n=18569)					Second stage (n=22318)					Combined (n=40887)				
SNPID	CHR	position	coded_all	noncoded_all	AF_coded_all	Effect*	SE	P-value	N	AF_coded_all	Effect*	SE	P-value	N	AF_coded_all	Effect*	SE	P-value	N
rs9905685	17	75,678,998	c	g	0.694	-0.010	0.003	2.68E-04	16677	0.677	0.003	0.005	5.57E-01	7506	0.689	-0.007	0.003	1.08E-02	24183
rs17138253	6	4,372,553	a	g	0.207	0.013	0.004	1.35E-04	6515	0.255	0.000	0.005	9.63E-01	7499	0.226	0.008	0.003	1.20E-02	14014
rs9320605	6	117,924,468	a	g	0.930	0.019	0.005	7.65E-05	16454	0.930	-0.012	0.008	1.43E-01	7506	0.930	0.011	0.004	1.22E-02	23960
rs8117359	20	23,593,826	c	g	0.880	0.016	0.004	9.56E-05	16384	0.870	-0.012	0.007	8.02E-02	7493	0.877	0.009	0.003	1.23E-02	23877
rs11638418	15	88,170,179	a	c	0.492	-0.011	0.003	3.09E-05	16675	0.502	0.004	0.004	3.82E-01	7504	0.495	-0.006	0.002	1.33E-02	24179
rs409896	13	112,448,339	t	g	0.147	-0.014	0.004	1.39E-04	16732	0.153	0.005	0.006	4.35E-01	7503	0.149	-0.008	0.003	1.46E-02	24235
rs17124445	10	110,158,286	c	g	0.977	0.046	0.010	8.64E-06	10527	0.976	-0.030	0.014	3.04E-02	7505	0.977	0.020	0.008	1.47E-02	18032
rs4600301	12	31,675,949	c	g	0.469	-0.010	0.003	7.01E-05	16677	0.460	0.003	0.004	5.37E-01	7494	0.466	-0.006	0.002	1.82E-02	24171
rs4856024	3	76,046,376	a	g	0.165	-0.013	0.004	1.78E-04	14794	0.161	0.003	0.006	5.62E-01	7472	0.164	-0.008	0.003	1.84E-02	22266
rs2050366	10	110,136,507	a	t	0.027	-0.037	0.009	4.62E-05	12357	0.021	0.033	0.015	2.27E-02	7505	0.025	-0.018	0.008	2.19E-02	19862
rs6436304	2	222,816,643	a	g	0.431	-0.011	0.003	3.65E-05	16677	0.428	0.005	0.004	2.10E-01	7495	0.430	-0.006	0.002	2.41E-02	24172
rs4931526	12	31,674,851	t	g	0.531	0.010	0.003	8.23E-05	16677	0.536	-0.003	0.004	4.24E-01	7494	0.533	0.006	0.002	2.41E-02	24171
rs6469257	8	110,471,632	t	g	0.447	-0.012	0.003	1.48E-04	4619	0.458	0.008	0.004	7.60E-02	7503	0.451	-0.006	0.002	2.57E-02	12122
rs9504097	6	4,372,282	a	g	0.205	0.013	0.004	1.90E-04	6519	0.248	-0.003	0.005	5.64E-01	7499	0.221	0.007	0.003	2.78E-02	14018
rs1367410	2	222,813,896	a	c	0.511	0.010	0.003	1.13E-04	16679	0.517	-0.004	0.004	3.10E-01	7506	0.513	0.005	0.002	3.40E-02	24185
rs420863	21	40,367,204	t	c	0.596	-0.011	0.003	1.22E-05	16675	0.595	0.007	0.004	9.47E-02	7500	0.596	-0.005	0.002	4.27E-02	24175
rs1005273	16	2,585,966	a	g	0.337	0.029	0.007	1.15E-05	4619	0.439	-0.015	0.008	6.46E-02	3613	0.380	0.010	0.005	5.12E-02	8232
rs975795	16	61,652,183	t	c	0.491	-0.010	0.003	5.93E-05	16669	0.471	0.006	0.004	1.84E-01	7435	0.484	-0.005	0.002	5.19E-02	24104
rs1520049	3	20,475,712	t	c	0.589	0.011	0.003	3.65E-05	16677	0.589	-0.008	0.004	6.40E-02	7500	0.589	0.005	0.002	5.52E-02	24177
rs7332653	13	96,315,120	a	c	0.320	-0.010	0.003	1.50E-04	16677	0.329	0.009	0.005	5.89E-02	7506	0.322	-0.005	0.003	6.52E-02	24183
rs1520051	3	20,474,103	t	c	0.407	-0.010	0.003	9.64E-05	16676	0.412	0.008	0.004	6.39E-02	7500	0.409	-0.004	0.002	9.99E-02	24176
rs10134399	14	103,153,210	a	g	0.361	-0.011	0.003	4.30E-05	16679	0.362	0.015	0.004	3.52E-04	7466	0.361	-0.002	0.002	3.53E-01	24145
rs2274268	14	103,099,131	c	g	0.644	0.011	0.003	5.97E-05	16199	0.641	-0.016	0.004	2.69E-04	7466	0.643	0.002	0.003	3.71E-01	23665
rs10098815	8	103,120,674	a	g	0.019	-0.246	0.055	9.24E-06	2144	0.198	0.006	0.010	5.57E-01	3401	0.192	-0.002	0.010	8.12E-01	5545

*The effect column shows the beta for difference between the natural log transformed on- and off-treatment LDL-C levels adjusted for natural log transformed off-treatment LDL-C, age, sex, and study specific covariates. Betas and p-values were generated using linear regression analysis. The beta reflects the fraction of differential LDL-C lowering in carriers vs. non-carriers of the SNP, a negative beta indicates a better statin response (stronger LDL-C reduction), a positive beta a worse statin response.

Supplementary Table 6: Additional genotyping of six SNPs in the Scandinavian participants of the ASCOT study

Chr	Position	SNP (gene)	Coding Allele	Phase	N	Freq coding allele	Beta*	SE	P-value
4	41185212	rs7696430	A	First stage	16,751	0.157	0.018	0.003	1.95x10^{-7}
		(*LIMCH1*)		Second stage + ASCOT	8,874	0.158	0.002	0.005	0.735
				All combined	25,625	0.157	0.014	0.003	8.38x10^{-8}
4	154871478	rs981844	A	First stage	18,169	0.747	0.011	0.002	9.36x10^{-6}
		(*RNF175*)		Second stage + ASCOT	8,820	0.749	-0.001	0.005	0.764
				All combined	26,989	0.747	0.009	0.002	9.67x10^{-7}
5	39090014	rs13166647	A	First stage	1,249	0.979	0.253	0.046	4.50x10^{-8}
		(*RICTOR*)		Second stage + ASCOT*	1,331	0.974	-2.52x10^{-5}	0.028	0.999
				All combined	2,580	0.976	0.068	0.024	0.004
5	38987383	rs13172966	T	First stage	1,249	0.979	0.251	0.046	5.33x10^{-8}
		(*RICTOR*)		Second stage + ASCOT*	1,378	0.968	0.001	0.026	0.985
				All combined	2,627	0.971	0.061	0.023	0.007
13	95098873	rs12428035	T	First stage	18,594	0.111	-0.015	0.003	9.52x10^{-6}
		(*DZIP1*)		Second stage + ASCOT	8,873	0.107	-0.006	0.006	0.325
				All combined	27,467	0.110	-0.013	0.003	8.36x10^{-7}
15	78977587	rs11638450	T	First stage	16,024	0.365	-0.015	0.003	1.51x10^{-5}
		(*KIAA1199*)		Second stage + ASCOT	8,808	0.395	-0.007	0.004	0.060
				All combined	24,832	0.376	-0.012	0.002	3.21x10^{-7}

*Beta for difference between the natural log transformed on- and off-treatment LDL-C levels adjusted for natural log transformed off-treatment LDL-C, age, sex, and study specific covariates. Betas and p-values were generated using linear regression analysis. A negative beta indicates a better statin response (stronger LDL-C reduction), a positive beta a worse statin response.

[#] SNPs from *RICTOR* which were only available within two first stage studies and within the ASCOT Scandinavians

Supplementary Table 7: Effect of genotype on post-treatment LDL-C with and without correction for measurement noise in baseline LDL-C in the CARDS (n=1194) study

CHR	SNP	Position	Beta adjusted for baseline LDL-C but uncorrected for measurement noise	Beta adjusted for baseline LDL-C and corrected for measurement noise
1	rs12740374	109619113	-0.0047	-0.0015
1	rs646776	109620053	-0.0054	-0.0019
4	rs7696430	41185212	0.0165	0.0142
6	rs2048327	160783522	0.0040	0.0026
6	rs11751605	160883220	0.0112	0.0099
6	rs10455872	160930108	0.0344	0.0276
12	rs2900478	21260064	0.0044	0.0029
19	rs1531517	49934013	-0.0217	-0.0154
19	rs10402271	50021054	0.0138	0.0103
19	rs7359852	50027875	0.0145	0.0106
19	rs2927480	50029225	0.0159	0.0117
19	rs1985096	50038391	-0.0307	-0.0238
19	rs4803763	50049131	0.0165	0.0121
19	rs6857	50084094	0.0312	0.0263
19	rs2075650	50087459	0.0294	0.0245
19	rs445925	50107480	-0.0374	-0.0262
19	rs4420638	50114786	0.0629	0.0549

Betas and p-values were generated using linear regression analysis.

Supplementary Table 8: Interaction between LDL-C response and placebo or statin allocation in JUPITER

CHR	SNP	Position	Coded allele	Non coded allele	MAF	N	beta	SE	P-value	Interaction P-value
1	rs646776	109620052	C	T	0.271	6961	-0.032	0.00742	1.50E-05	$4.73x10^{-2}$
6	rs10455872	160930107	G	A	0.053	6961	0.135	0.01414	2.04E-21	$1.89x10^{-9}$
12	rs2900478	21260063	A	T	0.173	6961	0.042	0.00866	1.43E-06	$1.74x10^{-4}$
19	rs445925	50107479	A	G	0.157	6961	-0.109	0.00914	9.93E-33	$7.71x10^{-10}$

Beta, SE and P-value refer to statistics from linear regression modeling the per allele change in the change in natural log (LDL-C) in response to statin treatment after 1 year of follow-up in JUPITER as described in **Online methods**. The interaction p-value refers to the significance of an SNP-by-placebo allocation interaction term in the same regression model.

Supplementary Table 9: Genome-wide conditional analysis with summary level data from combined meta-analysis using GCTA

			GIST results					Genome-wide Conditional Analysis				
**Chr**	**SNP**	**Position**	**freq**	**beta**	**se**	**p-value**	**n**	**bJ**	**bJ_se**	**pJ**	**LD_r**	**Gene**
1	rs12740374	109619113	0.223	-0.013	0.002	1.05E-09	38579	-0.014	0.002	2.82E-12	-0.029	*CELSR2*
3	rs909078	121846997	0.728	0.029	0.007	6.25E-05	4727	0.028	0.008	8.77E-04	0.023	*HGD*
4	rs981844	154871478	0.748	0.010	0.002	2.13E-07	25659	0.012	0.002	2.88E-07	-0.035	*RNF175*
5	rs6884324	62213017	0.095	-0.022	0.005	4.72E-05	10137	-0.024	0.005	8.38E-06	-0.032	*ISCA1L-HTR1A*
6	rs2048327	160783522	0.621	-0.014	0.003	8.76E-09	24185	-0.007	0.002	6.75E-04	0.296	*SLC22A3*
6	rs10455872	160930108	0.920	-0.052	0.004	7.41E-44	31038	-0.052	0.004	1.43E-40	0.013	*LPA*
9	rs7865666	4368591	0.658	0.010	0.003	2.35E-04	10115	0.012	0.003	3.44E-04	0.014	*GLIS3-SLC1A1*
9	rs1930096	110393856	0.098	-0.027	0.006	8.47E-06	10285	-0.027	0.006	4.42E-06	0.001	*LOC100128657*
10	rs2111326	101352526	0.503	-0.010	0.003	5.82E-04	20286	-0.011	0.002	3.73E-06	0.003	*NKX2-3-SLC25A28*
12	rs17813281	68002012	0.125	-0.018	0.005	2.11E-04	16574	-0.021	0.004	2.26E-07	-0.008	*LOC729409-LYZ*
14	rs17091112	55732898	0.937	0.022	0.005	1.31E-05	14064	0.024	0.005	1.08E-05	-0.006	*PELI2*
19	rs4803763	50049131	0.279	0.021	0.003	5.65E-14	20309	0.018	0.003	2.11E-08	0.108	*PVRL2*
19	rs6857	50084094	0.136	0.022	0.003	1.75E-14	34544	0.020	0.003	1.03E-10	-0.059	*Apoe4* tagger
19	rs445925	50107480	0.108	-0.051	0.005	8.52E-29	17742	-0.048	0.005	2.57E-23	0.000	*Apoe2* tagger

Abbreviations: bJ, beta in conditional analysis; bJ_se, SE in conditional analysis; pJ, P-value in the conditional analysis; LD_r, D'prime between the top SNP and other SNPS.

Supplementary Table 10: GIST GWAS results of previous identified loci associated with statin response

Chr	SNP	EAF	Beta*	SE	P-value	Sample size	Gene	Reference
1	rs11583680	0.133	-0.004	0.004	0.358	16751	*PCSK9*	[2]
4	rs1481012	0.885	-0.001	0.004	0.858	16748	*ABCG2*	[3]
5	rs10474433	0.656	-0.003	0.002	0.259	18581	*HMGCR*	[2]
5	rs17671591	0.344	0.003	0.002	0.259	18560	*HMGCR*	[2]
5	rs2303151	0.055	-0.004	0.005	0.431	18284	*HMGCR*	[4]
6	rs20455	0.647	-0.002	0.003	0.474	16693	*KIF-6*	[5]
10	rs2002042	0.260	-0.006	0.003	0.028	16746	*ABCC2*	[6]
14	rs8014194	0.259	0.001	0.003	0.740	16728	*CLMN*	[7]
19	rs11668477	0.816	0.008	0.003	0.019	16750	*LDLR*	[3]

*Beta for difference between the natural log transformed on- and off-treatment LDL-C levels adjusted for natural log transformed off-treatment LDL-C, age, sex, and study specific covariates. Betas and p-values were generated using linear regression analysis. A negative beta indicates a better statin response (stronger LDL-C reduction), a positive beta a worse statin response.
Abbreviations: EAF, effect allele frequency; SE, standard error

Supplementary Note 1. Participating Randomized controlled trials*Anglo-Scandinavian cardiac Outcomes Trial (ASCOT)*

Of 19,342 hypertensive patients (40–79 years of age with at least three other cardiovascular risk factors) who were randomized to one of two antihypertensive regimens in ASCOT, 10,305 with non-fasting TC concentrations of 6.5 mmol/l or less (measured at the non-fasting screening visit) had been randomly assigned additional atorvastatin 10 mg or placebo. These patients formed the lipid-lowering arm of the study. For this genome-wide study only a proportion of United Kingdom, Irish, Sweden, Norway, Finland and Denmark consented to participate. On UK and Irish GWAS was available but those from Scandinavian countries were involved in wet bench SNP replication studies. In both the GWAS and replication resources there were two subpopulations from ASCOT included. The first subpopulation included individuals randomized to 10 mg atorvastatin in whom pre-treatment LDL-C was measured at the (fasting) randomization visit and on-treatment LDL-C was calculated as the simple average of measures at the (fasting) visits 6 months and 12 months post-randomization. LDL- C was estimated using the Friedewald equation. Following the end of the randomization phase, there was an observational period. The second subpopulation included all individuals not originally randomized to 10 mg atorvastatin (i.e., those randomized to placebo and those not eligible for the LLA) who were subsequently prescribed atorvastatin 10 mg. For these individuals, pre-treatment LDL- C was defined as the measurement on the last visit before or equal to date of starting atorvastatin, and on-treatment LDL- C was defined as the measurement taken from the first visit after date of starting atorvastatin.

Collaborative Atorvastatin Diabetes Study (CARDS)

Methods in CARDS have been described previously [8, 9]. In brief, 2838 patients with Type 2

diabetes and no previous CVD were randomized to receive either placebo or atorvastatin 10mg once daily and followed for a median of 3.7 years. Allocation was double blinded. Mean serum LDL- C concentration during baseline visits prior to randomization had to be = 4.14mmol/L (160 mg/dl) and serum triglycerides = 6.78mmol/L (600mg/dl). After randomization, total cholesterol, HDL-C, and triglycerides were measured at one, two, and three months and 6 monthly thereafter. Patients attended after an overnight fast. LDL-c was calculated with the Friedewald formula [10] or, if serum triglycerides exceeded 4·0 mmol/L, by removing VLDL by ultracentrifugation then measuring the change in infranatant cholesterol content when LDL was removed by precipitation of apolipoprotein B-containing lipoproteins. For this genome-wide study the analyses were restricted to those randomized to atorvastatin and the mean of two pre-treatment LDL- C measurements was used as the baseline LDL- C and a weighted average of five post-randomization values within the first year post-randomization was the outcome measure or "on treatment LDL- C", with weights (0.6 for month 1 and then 0.1 for measurements at 2, 3, 6 and 12 months). The genotyping methods in CARDS have been described in details elsewhere [11].

Cholesterol and Atherosclerosis Pharmacogenetics (CAP)[1]

The trial involved 944 healthy volunteers, 609 of whom were Caucasian [12]. Participants were aged 30 and above, who received open label 40 mg simvastatin daily for 6 weeks. They were recruited from two clinical sites located in Los Angeles and San Francisco, California, respectively. Screening criteria included serum total cholesterol levels of 4.14-10.36 mmol/L (160-400 mg/dL). Lipids, including LDL-C determined by the Friedewald formula, were measured

[1] CAP was designed as a pharmacogenetics study and therefore not a randomized placebo-controlled trial

twice prior to treatment (at screening and after a two-week run-in period) and twice on treatment (4 and 6 weeks), and the averages were used for each time point. Human subject approvals were obtained at all participating institutions and all participants signed statements of informed consent. In total, 591 subjects with both lipids and DNA data were available for analysis. Discovery genotyping was performed for half of the subjects using beadchip technology (HumanHap300 BeadChip , Illumina Inc. San Diego CA) for whole-genome genotyping of 314,621 tagSNP markers derived from the International HapMap Project. Genome-wide genotyping was performed on the remaining half of the samples using the Illumina HumanCNV610-Quad beadchip containing 620,901 tagSNPs. SNPs with MAF < 1% and proper information <0.30 (obtained by SNPTEST) were excluded from analysis. Imputation was performed using BIMBAM v0.99 with reference to HapMap CEU using release 23, build 36.

PRavastatin INflammation CRP Evaluation study (PRINCE)

Participants were Caucasians, aged 21 and older, who received 40 mg daily pravastatin for 12 weeks [13]. They were enrolled from 1143 sites representing 49 states and the District of Columbia, with no single site enrolling more than 4 individuals. Recruitment criteria included either an LDL-Cl concentration ≥3.5 mmol/L (>135 mg/dL) or a history of myocardial infarction, stroke, or coronary revascularization regardless of baseline LDL-C. Lipid measurements including LDL-C determined by the Friedewald formula were obtained once prior to treatment and once following 12 weeks of treatment. Human subjects approvals were obtained at all participating institutions and all participants signed statements of informed consent. In total, 1348 participants had DNA available for whole genome-wide association analysis. Genotyping and imputation were performed using the same platforms and procedures as for CAP i.e., half of the samples with each of the Illumina platforms).

PROspective Study of Pravastatin in the Elderly at Risk (PROSPER)

All data come from the PROspective Study of Pravastatin in the Elderly at Risk (PROSPER). A detailed description of the study has been published elsewhere [14, 15]. PROSPER was a prospective multicenter randomized placebo-controlled trial to assess whether treatment with pravastatin diminishes the risk of major vascular events in elderly. Between December 1997 and May 1999, we screened and enrolled subjects in Scotland (Glasgow), Ireland (Cork), and the Netherlands (Leiden). Men and women aged 70-82 years were recruited if they had pre-existing vascular disease or increased risk of such disease because of smoking, hypertension, or diabetes. A total number of 5,804 subjects were randomly assigned to pravastatin or placebo. A large number of prospective tests were performed including Biobank tests and cognitive function measurements. A whole genome wide screening has been performed in the sequential PHASE project with the use of the Illumina 660K beadchip [16]. Of 5,763 subjects DNA was available for genotyping. Genotyping was performed with the Illumina 660K beadchip, after QC (call rate <95%) 5,244 subjects and 557,192 SNPs were left for analysis. These SNPs were imputed to 2.5 million SNPs based on the HAPMAP built 36 with MACH imputation software.

Plasma lipids and lipoproteins were measured twice during the screening phase, i.e. at the beginning and end of the single-blind, placebo "run-in" phase according to the standardized Lipid Research Clinics protocol. Baseline LDL-C levels were taken as the average of these 2 determinations prior to randomization to statin treatment. During follow-up, plasma lipids and lipoproteins were measured after 3, 6, 12, 24, and 36 months. Total cholesterol (TC), HDL cholesterol, and triglycerides were assessed after an overnight fast, LDL-C was calculated by the Friedewald formula, as previously described [14].

Treating to New Targets (TNT)

The design of the TNT trial has been described in details elsewhere [17]. In brief, 10 001 patients with stable coronary heart disease (CHD) and LDL-C levels <130 mg/dL (3.4 mmol/L) were randomly assigned to receive either 10 or 80 mg of atorvastatin per day and were followed up for a median of 4.9 years. Mean LDL-C levels during treatment were 101 mg/dL (2.6 mmol/L) and 77 mg/dL (2.0 mmol/L) for the 10- and 80-mg groups, respectively. At screening, LDL-C, high-density lipoprotein cholesterol (HDL-C), triglycerides (TG), and total cholesterol were measured in all subjects in a fasting state. In addition, blood pressure and body mass index as well as other standard blood chemistries were measured. All laboratory tests were performed at a central laboratory (Medical Research Laboratories, Highland Heights, Ky) certified by the National Heart, Lung, and Blood Institute/Centers for Disease Control Part III Program. These were repeated 4 weeks later, at randomization, 3 and 6 months post randomization, and annually thereafter.

After approval by the institutional review committee, informed consent for genetic analysis was sought on entry into the trial and 5966 DNA samples were obtained from consenting individuals. A subset was chosen for whole-genome analysis based on the cardiovascular events during the course of the trial and those individuals were matched 3:1 with controls based on age, gender, treatment arm, smoking, diabetes, hypertension, baseline lipid values, baseline glucose levels, and screening LDL-C. The Perlegen 322K array genotyping array was used to perform genome-wide genotyping. Samples and SNPs will call rate equal or under 98% were removed prior to the analyses. IMPUTE 2 (v. 2.1.0) and GTOOL (v.0.6.6) were used to impute additional SNPs which were analyzed for their association with LDL response with PLINK (v.1.07)

Supplementary Note 2: Participating Observational studies

Age, Gene/Environment Susceptibility-Reykjavik (AGES) study

The Reykjavik Study cohort originally comprised a random sample of 30,795 men and women born in 1907-1935 and living in Reykjavik in 1967. A total of 19,381 attended, resulting in 71% recruitment rate. Between 2002 and 2006, the AGES-Reykjavik study re-examined 5,764 survivors of the original cohort who had participated before in the Reykjavik Study [18]. Serum lipid levels were measured at baseline (AGES:2002-2006), and at a follow-up five years later (AGESII:2007-2001). Individuals were recruited in the same order. Of the 5,764 participants, 3,664 participants were randomly selected for the GWAS. Genotyping was undertaken using the HumanCNV370-Duo (Illumina) at the Laboratory of Neurogenetics, Intramural Research Program, at the National Institute of Aging, Bethesda, Maryland.

Atherosclerosis Risk in Communities (ARIC) study

The ARIC study is an ongoing population-based cohort of 15,792 predominantly Caucasian and African-American males and females aged 45-64 years at baseline and selected using probability sampling from four United States communities (Forsyth County NC, Jackson MS, suburban Minneapolis MN, and Washington County MD) [19]. Participants were recruited in 1987-1989 to examine cardiovascular and pulmonary disease, patterns of medical care, and disease variation over time. Standardized physical examinations and interviewer-administered questionnaires were conducted at baseline (1987-1989), and at three triennial follow-up examinations (1990-1998). Eligible participants for this effort were from the NC, MN, and MD field centers, as only Caucasian participants were examined in this analysis and the MS center only recruited African American participants.

Twelve-hour fasting total cholesterol and HDL were measured, lipids and lipoproteins were

measured as previously described at baseline and during the three triennial exams [20, 21]. LDL-C was calculated using the Friedewald formula.

The Affymetrix 6.0 genotype array was used to genotype n=669,450 SNPs that passed quality control (sample call rate ≥ 0.95; SNP call rate ≥ 0.90; SNP MAF filter ≥ 0.01, HWE p-value filter ≥ 10^{-5}). SNPs were imputed based on the HAPMAP build 36 with MACH v1.16 and analyses were performed using ProbABEL.

BioVU

BioVU is the nation's largest collection of DNA samples linked to a comprehensive, de-identified electronic medical record (EMR) [22, 23]. BioVU began in 2007 and accrues DNA samples via an opt-out model (the design and ethical principles of which have been described previously [22] primarily from outpatient visits. On September 3, 2013, the biobank contains 151,605 adults linked to de-identified mirror images of individual comprehensive EMRs. This database is scrubbed of all Health Insurance Portability Accountability Act (HIPAA) identifiers; e.g., if the name "John Smith" appears in the original record, its corresponding record in the synthetic derivative is permanently replaced with a tag [NAME AAA, BBB] to maintain the semantic integrity of the text.

Per BioVU policy, all projects utilizing BioVU samples are required to redeposit their genotyping results into the BioVU databases for reuse by other investigators. Of note, 29,876 individuals have genome-wide SNP data available on September 3, 2013, and were among the available population used for this study.

Plasma lipid data is extracted from linked de-identified EMRs. Before Treatment LDL-C is defined as median LDL-C of 18 months window before first ever statin mentioned in the EMRs. After Treatment LDL-C is defined as median LDL-C of 18 months window after first ever statin

mentioned in the EMRs, with right censoring within the 18 months window at the first drug or dose changes. LDL-C change is calculated by Before Treatment LDL-C minus After Treatment LDL-C. Subjects with positive LDL-C change and genome-wide SNP data available are included in the final analyses. Statin exposures were derived from electronic prescribing tools and use of the MedEx natural language processing tool, which extracts medication references from narrative text [24]. When using MedEx, we apply heuristic rules to identify patients truly receiving medications and filter out adverse events, medication discussions, and other non-prescription events.

Cardiovascular Health Study (CHS)

The CHS is a population-based cohort study of risk factors for CHD and stroke in adults ≥65 years conducted across four field centers [25]. The original predominantly Caucasian cohort of 5,201 persons was recruited in 1989-1990 from random samples of the Medicare eligibility lists; subsequently, in 1992-1993, an additional predominantly African-American cohort of 687 persons was enrolled for a total sample of 5,888. DNA was extracted from blood samples drawn on all participants at their baseline examination. In 2007-2008, genotyping was performed at the General Clinical Research Center's Phenotyping/Genotyping Laboratory at Cedars-Sinai using the Illumina 370CNV BeadChip system on 3980 CHS participants who were free of CVD at baseline, consented to genetic testing, and had DNA available for genotyping. Because the other cohorts were predominantly white, the African American participants were excluded from this analysis. Thus, for this analysis, the study sample is limited to European ancestry participants who used statins during follow up with available genotype data as well as on- and off-treatment lipid measures.

In CHS, the following exclusions were applied to identify a final set of 306,655 autosomal SNPs:

call rate < 97%, HWE P < 10-5, > 2 duplicate errors or Mendelian inconsistencies (for reference CEPH trios), heterozygote frequency = 0, SNP not found in HapMap. Imputation was performed using BIMBAM v0.99 with reference to HapMap CEU using release 22, build 36 using one round of imputations and the default expectation-maximization warm-ups and runs.

Plasma lipids and lipoproteins were measured several times during follow-up; low-density lipoprotein cholesterol (LDL-C) measurements are available from baseline, year 3, and year 18. Subjects came to the clinic after an overnight fast, and blood was obtained on their arrival at the clinic. Samples were shipped weekly, on dry ice, to the CHS Central Blood Laboratory at the University of Vermont, where all analyses were performed. Plasma total cholesterol and triglyceride (TG) were measured by enzymatic methods on an Olympus Demand System (Olympus Corp., Lake Success, N.Y.). High density lipoprotein cholesterol (HDL-C)was measured by an enzymatic method after precipitation of apo-lipoprotein B-containing lipoproteins with dextran sulfate/magnesium sulfate. Low-density lipoprotein cholesterol (LDL-C) was calculated according to the Friedewald equation for individuals whose serum TG was <4.51mmol/l [26].

Framingham Heart Study (FHS)

The methods for recruitment and clinical covariate collection have been described previously for the original Framingham Heart Study cohort (5,209 participants ascertained systematically from two-thirds of the households in the town of Framingham, MA, beginning in 1948)[27], the Framingham Heart Study Offspring cohort (5,124 children of the original cohort, and spouses of those children, beginning in 1972)[28], and the Third Generation cohort (4,095 children of the Offspring cohort, beginning in 2002)[29]. The current study was conducted in 665 participants recruited in the Offspring Cohort from Exam 4 (1987-1991) through Exam 8 (2005-2008). Low Density lipoprotein (LDL) was calculated with the Friedewald formula for those individuals with

triglycerides less than 400 based on fasting measures of plasma total cholesterol, high density lipoprotein and triglycerides collected at each exam. To evaluate the effect of lipid lowering therapy, we selected the first exam at which a person reported using lipid lowering medication. It is assumed that most of the therapy during this time was statin, but we did not have specific data on which medications were used except for Exam 8. Then we used the LDL-C measure from that examination and the previous exam (approximately 4 years before) to calculate the logarithm of LDL-C before lipid lowering treatment minus the logarithm of LDL-C after the beginning use of lipid lowering treatment. This trait was analyzed in mixed effects linear regression models (accounting for familial relationships) for all genetic variants in the CEU sample of the Phase 2 HapMap Release 22. We adjusted for sex, age, time between the LDL-Cmeasurements and Principal Component 7 to control for population substructure. Genotyping was conducted for the SNP Health Association Resource (SHARe) project (http://www.ncbi.nlm.nih.gov/projects/gap/cgi-bin/study.cgi?study_id=phs000007.v20.p8) using the Affymetrix 500K mapping array (250K Nsp and 250K Sty arrays) and the Affymetrix 50K supplemental gene focused array on a total of 9,274 individuals from all three cohorts. To evaluate population substratification, we conducted principal component analyses using EIGENSTRAT [30] on the genotypes from 882 unrelated participants. We estimated the first 10 principal components and applied the loadings of these components to all genotyped participants. Finally, we evaluated whether any of these principal components were associated with the difference in the logarithms of LDL before and after lipid lowering initiation. Only Principle Component 7 was associated with LDL-C and was thus included in the regression model. Genotyping resulted in 503,551 SNPs with successful call rate >95% and HWE P>1.0x10-6 in 8,481 individuals with call rate >97%. Imputation of 2,543,887 autosomal SNPs in HapMap release 22, CEU sample was conducted using the algorithm implemented in MACH (version

1.0.15). From a total of 534,982 genotyped autosomal SNPs in Framingham, 378,163 SNPs were used in imputation after filtering out 15,586 SNPs (HWE $P<1.0x10^{-6}$), 64,511 SNPs (missingness >0.03), 45,361 SNPs (mishap $P<1.0x10^{-9}$), 4,857 SNPs (>100 Mendel errors), 67,269 SNPs (frequency <0.01), 2 SNPs (due to strand issues upon merging data with HapMap), and a further 13,394 SNPs that were not present on HapMap. We used 200 biologically unrelated participants to estimate the parameters of the imputation model and subsequently applied the estimated parameters to obtain imputed SNPs for all 8,481 participants. The Framingham Heart Study, including genetic association studies of Framingham phenotypes, was approved by the institutional review boards of Boston University and the National Institutes of Health. All participants provided written informed consent.

Genetics of diabetes Audit and Research (GoDARTS)

We performed an observational study using data from the Genetics of Diabetes Audit and Research (GoDARTS) database, which has been described previously [31, 32]. This includes detailed clinical information on ~15,000 patients with and without diabetes who have provided consent to link their genetic information with their healthcare record in Tayside Scotland from 1990 to present, including demographic data, all prescriptions dispensed from Tayside pharmacies, all biochemistry data from the region-wide clinical laboratory, Scottish Morbidity Records (SMR), detailing International Classification of Disease (ICD) coding for hospital admissions and data from the General Registrar's Office detailing date and cause of death.

Study Population: The study population included all subjects within the Go-DARTS cohort, who were resident in Tayside during the study period 1st January 1990 to 31st January 2010.

Study criteria: To assess treatment response, individuals in the study were required to have had at least one on-treatment LDL-C/total cholesterol (TC) measure. The biochemistry laboratories

in Tayside use the Friedewald equation to calculate LDL-C which is not directly measured. In addition, patients must have had at least two statin prescriptions.

Definition of study period: Duration of statin therapy was defined as the period between first (index date) and last statin prescription. However, if patients were on a lipid-lowering drug other than statins (defined as all other drugs in BNF chapter 2.12) at index date and this drug was subsequently stopped then the study period was censored at this point. In addition, if a lipid-lowering drug was initiated during statin exposure then the study was also censored at this point.

Definition of statin lipid-lowering response outcome: Pre-treatment LDL-C was defined as closest measure before statin initiation and on-treatment LDL-C was defined as the lowest LDL-C achieved whilst on statins. Where pre-treatment LDL-C was not available values were estimated using multiple imputation methods (PROC MI) in SAS, which was described and validated previously [33].

GWAS of statin response was performed in two non-overlapping sub groups of the GoDARTS population as described below. Summary results were provided for meta-analysis of both tranches separately.

GoDARTS WTCCC2 genotyping (GoDARTS1): Samples were genotyped at Affymetrix's service laboratory on the Genome-Wide Human SNP Array 6.0. For all samples passing Affymetrix's laboratory quality control, raw intensities were renormalized within collections using CelQuantileNorm. These normalized intensities were used to call genotypes with an updated version of the Chiamo software adapted for Affymetrix 6.0 SNP data.

Genotype data quality control was via the protocol that was established for the WTCCC2 studies [34]. The GWAS data was imputed to Hapmap 2.2 using IMPUTE2 with the HapMap 2.2 CEU reference panel and an additional panel of the WTCCC2 control group that had been directly

typed on both the Illumina and the Affymetrix platforms. This provided 3734 samples on 2.7 million SNPs. The imputed dataset was analyzed in SNPTEST. SNPs were removed if the missing rate was above 10% and the estimated minor allele frequency was below 1%. SNPs that significantly deviated from Hardy Weinberg Equilibrium ($p<1x10^{-6}$) were also removed and the final data set consisted of 705,125 autosomal SNPs.

GoDARTS IMI-SUMMIT genotyping (GoDARTS2): A total number of 3499 patients were genotyped with the Illumina HumanOmniExpress array. SNPs with a call rate lower than 98% or significantly deviate from Hardy Weinberg Equilibrium ($p<1x10^{-6}$) or minor allele frequency less than 0.001 were removed from further analysis. Samples with gender discrepancy, a call rate lower than 98% and heterozygosity being more than 3 standard deviations from the sample average were removed from further analysis. Where there was a pairwise IBD>0.125 the sample with lower call rate was removed from further analysis. We also performed PCA analysis with EIGENSTRAT on the default parameters to remove samples of ancestry outliers. A total number of 62914 autosomal SNPs on 3299 samples passed this QC and were used for imputation.

The SNPs were aligned to NCBI genome build 36.3 and phased with SHAPEIT2. The best estimate haplotypes were then used for imputation with IMPUTE2 with the HapMap2 CEU panel as reference. Post imputation filter was applied to SNPs with info<0.3 when SNPTEST was used for the statin response association tests on 2158 patients with defined LDL-C treatment outcome.

Health Aging and Body Composition (Health ABC) Study

Health ABC is a NIA-sponsored ongoing cohort study of the factors that contribute to incident disability and the decline in function of healthier older persons, with a particular emphasis on changes in body composition in old age. Health ABC enrolled well-functioning, community-dwelling black (n=1281) and white (n=1794) men and women aged 70-79 years between April

1997 and June 1998. Participants were recruited from a random sample of white and all black Medicare eligible residents in the Pittsburgh, PA, and Memphis, TN, metropolitan areas. The key components of Health ABC include a baseline exam, annual follow-up clinical exams, and phone contacts every 6 months to identify major health events and document functional status between clinic visits. GWAS data are available from 1663 white participants.

Genomic DNA was extracted from buffy coat collected using PUREGENE® DNA Purification Kit during the baseline exam. Genotyping was performed by the Center for Inherited Disease Research (CIDR) using the Illumina Human1M-Duo BeadChip system. Samples were excluded from the dataset for the reasons of sample failure, genotypic sex mismatch, and first-degree relative of an included individual based on genotype data. Genotyping was successful for 1,151,215 SNPs in 1663 unrelated. Imputation was done for the autosomes using the MACH software version 1.0.16. SNPs with minor allele frequency ≥ 1%, call rate ≥97% and HWE p≥10-6 were used for imputation. HapMap II phased haplotypes were used as reference panels. For EAs, genotypes were available on 914,263 high quality SNPs for imputation based on the HapMap CEPH reference panel (release 22, build 36). A total of 2,543,887 SNPs in EAs are available for analysis. The European Ancestry participants in Health ABC underwent a second phase of imputation based on the June 2010 release of the 1000 Genomes Project haplotypes based on the CEU sample subset. Two-step imputation using MACHv1.0.16 was implemented yielding a gross total of 6,858,242 SNPs prior to quality filtering.

Heart and Vascular Health (HVH) Study

The setting for this study was Group Health (GH), a large integrated health care system in western Washington State. Data were utilized from an ongoing case-control study of incident myocardial infarction (MI) and stroke cases with a shared common control group. Methods for

the study have been described previously and are briefly summarized below [35-37]. The study was approved by the human subjects committee at GH, and written informed consent was provided by all study participants.

All study participants were GH members and aged 30-79 years. MI and stroke cases were identified from hospital discharge diagnosis codes and were validated by medical record review. Controls were a random sample of GH members frequency matched to MI cases on age (within decade), sex, treated hypertension, and calendar year of identification. The index date for controls was a computer-generated random date within the calendar year for which they had been selected. For MI cases, the index date was the date of admission for the first acute MI. Participants were excluded if they were recent enrollees at GHC, had a history of prior MI or stroke, or if the incident event was a complication of a procedure or surgery.

Eligibility and risk factor information were collected by trained medical record abstractors from a review of the GH medical record using only data available prior to the index date and through a telephone interview. Medication use was ascertained using computerized GH pharmacy records. A venous blood sample was collected from all consenting subjects, and DNA was extracted from white blood cells using standard procedures.

Genotyping was performed at the General Clinical Research Center's Phenotyping/Genotyping Laboratory at Cedars-Sinai using the Illumina 370CNV BeadChip system. Genotypes were called using the Illumina BeadStudio software. Samples were excluded from analysis for sex mismatch or call rate < 95%. The following exclusions were applied to identify a final set of 301,321 autosomal SNPs: call rate < 97%, HWE P < 10-5, > 2 duplicate errors or Mendelian inconsistencies (for reference CEPH trios), heterozygote frequency = 0, SNP not found in HapMap, inconsistencies across genotyping batches. Imputation was performed using BIMBAM with reference to HapMap CEU using release 22, build 36 using one round of imputations and

the default expectation-maximization warm-ups and runs.

Plasma lipids and lipoproteins were measured over the course of general care at Group Health and were obtained from the outpatient medical record and/or Group Health laboratory database.

Multi-Ethnic Study of Atherosclerosis (MESA)

The Multi-Ethnic Study of Atherosclerosis (MESA) is a study of the characteristics of subclinical cardiovascular disease (disease detected non-invasively before it has produced clinical signs and symptoms) and the risk factors that predict progression to clinically overt cardiovascular disease or progression of the subclinical disease. MESA researchers study a diverse, population-based sample of 6,814 asymptomatic men and women aged 45-84. Thirty-eight percent of the recruited participants were white, 28 percent African-American, 22 percent Hispanic, and 12 percent Asian, predominantly of Chinese descent [38]. Participants were recruited from six field centers across the United States and followed-up three times with an average time period of follow-up of 2 years between each visit. Data from four visits (exam1 to exam4) was used for the analysis. Subjects on treatment at the time point of follow-up visit and off treatment at the previous visit were qualified for inclusion. Phenotype (lipids measures before and after statin treatment) and genotype data were available for 360 Caucasian subjects. The tenets of the Declaration of Helsinki were followed and institutional review board approval was granted at all MESA sites. Written informed consent was obtained from each participant.

Genotyping was performed using the Affymetrix Genome-Wide Human SNP Array 6.0. IMPUTE version 2.1.0 was used to perform imputation for the MESA Caucasian participants (chromosomes 1-22) using HapMap Phase I and II - CEU as the reference panel (release #24 -

NCBI Build 36 (dbSNP b126)). SNPs with MAF less than 0.02 or HWE p value less than 0.001 were removed from the analysis.

Supplementary Note 3: Studies participating in second stage

The Heart Protection Study (HPS)

Between 1994 and 1997, 20536 men and women aged 40–80 years were recruited from 69 collaborating hospitals in the UK (with ethics committee approval). Participants were eligible for inclusion if they had non-fasting blood total cholesterol concentrations of at least 3.5 mmol/L (135 mg/dL) and either a previous diagnosis of coronary disease, ischaemic stroke, other occlusive disease of noncoronary arteries, diabetes mellitus, or (if men 65 years or older) treated hypertension. Patients were not on statin therapy at entry into the study. At the screening visit, all participants provided written consent and began a pre-randomization 'run-in' phase involving 4 weeks of placebo followed by 4-6 weeks of 40 mg simvastatin daily, after which fully compliant individuals were randomly allocated to 40 mg simvastatin daily or matching placebo for about 5 years. A non-fasting blood sample was taken at screening (i.e. before starting any statin therapy) and at the end of run-in (i.e. while on 40 mg simvastatin daily). The pre-specified primary outcome for assessing the effect of statin therapy in different subgroups was the first occurrence after randomization of an incident major vascular event

(defined as non-fatal MI or coronary death, coronary or non-coronary revascularizations, or any stroke). In the central laboratory, Beckman autoanalysers used standard spectrophotometric enzymatic methods to measure LDL-C directly. Further details of the Heart Protection Study are reported elsewhere [39].

For genotyping a random selection of 4000 self-reported Caucasians with lipids and other biomarker measurements were selected, and genotypes measured using the Illumina 610K Quad panel. Genome-wide data were available for 3,895 individuals after quality control exclusions (see Supplementary Table 3). In addition, custom I.PLEX panels were used to measure 31 lipid response candidate SNPs of interest in the remaining 14,810 participants in the Heart Protection Study with available DNA, yielding directly measured genotypes in up to 18,705 individuals. Further details of the genotyping undertaken in the Heart Protection Study are reported elsewhere [6].

Justification for the Use of statins in Prevention: an Intervention Trial Evaluating Rosuvastatin (JUPITER) population

The study population was derived from JUPITER, an international, randomized, placebo-controlled trial of rosuvastatin (20mg/day) in the primary prevention of cardiovascular disease conducted among apparently healthy men and women with LDL-C < 130 mg/dL and hsCRP ≥ 2 mg/L [40]. Individuals with diabetes or triglyceride concentration >500mg/dL were also excluded. Approximately 71.2% of JUPITER participants had European ancestry among whom 71.4% provided DNA and consent for genetic analysis. The present analysis includes only individuals with genotype information who had verified European ancestry (see below) and who were deemed compliant with the study protocol as judged by on the basis of pill counts and reported absence of non-trial statin use, i.e. information that was independent of LDL-C reduction. After

applying these restrictions, 3401 statin- and 3356 placebo-allocated participants remained for analysis.

Per study protocol, all JUPITER participants had standard lipid measurements made in a core laboratory facility prior to randomization and again after one year of placebo or rosuvastatin treatment. LDL cholesterol was calculated by the Friedwald equation from measured concentrations of total cholesterol, triglycerides, and HDL cholesterol.

Genotyping in the JUPITER population was performed using the Omni 1M Quad platform (Illumina, San Diego) [3]. Briefly, raw genotype intensity data were reduced to genotype calls using the Illumina Genome Studio (v. 1.6.2) software (Illumina, San Diego). SNP clusters were initially defined automatically using data from the JUPITER sample and SNPs failing quality metrics were visually inspected and manually clustered again or removed as appropriate. After these procedures, 99.71% of the loci yielded successful genotype information. In the final data used for analysis, samples were retained if >98% of the SNPs had successfully genotype, while SNPs were retained if the updated clusters met quality standards and the genotyping was successful in >90% of the samples. Of JUPITER participants with self-reported European ancestry who provided consent for genetic analysis, over 99% or 8,749 had successful genotyping and verification of their ancestry with identity-by-state clustering using multi-dimensional scaling procedures in PLINK [41] applied to 1,067 ancestry informative SNPs from HapMap3. Among JUPITER participants with verified European ancestry, rs7412, which distinguishes the APOE E2 from E3/E4 genotypes deviated from Hardy-Weinberg equilibrium, likely due to the ascertainment criteria related to LDL-C levels in JUPITER. This SNP was included in the final data only after manual inspection of genotyping clusters. Sub-European ancestral stratification was estimated using the principal component approach in EIGENSTRAT [30]. Genotypes for SNPs in the 1000 genomes pilot data (release 2010-03) were imputed with MaCH v. 1.0.16 [42].

Rotterdam study

The Rotterdam Study is a prospective population-based cohort study of chronic diseases in the elderly population. From 1990 to 1993, 7 983 inhabitants of the suburb Ommoord in Rotterdam, the Netherlands, aged 55 years or older, entered the Rotterdam Study (RS-I) and gave written informed consent. Ethical approval was obtained from the Medical Ethical Committee of the Erasmus Medical Center, Rotterdam, the Netherlands. Participants were invited between 1990 and 1993 and have been continuously followed since them. Medication prescription data were obtained from all seven fully computerized pharmacies in the Ommoord suburb. These pharmacies dispense the prescriptions of more than 99% of all participants. Information on all filled prescriptions from January 1st 1991 until June 1st 2008 was available and included information on the product name of the drug, the Anatomical Therapeutical Chemical code, the amount dispensed, the prescribed dosage regimen and the date of dispensing. Furthermore, in 2000, an extended cohort was enrolled, the Rotterdam Study II (RS-II). 3 011 inhabitants entered the study and have been continuously followed since then. Detailed information on design, objectives and methods of this study have been described before [43, 44].

Lipid measurements were obtained from all participants at each visits. Fasting total cholesterol, HDL-cholesterol and triglyceride levels were determined using enzymatic procedures (Hitachi Analyzer, Roche Diagnostics). The Friedewald equation was used to estimate LDL-C [10].

At baseline examination of the Rotterdam Study, blood was taken from which genomic DNA was extracted, using the salting-out method [45]. Microarray genotyping was performed in both Rotterdam Study cohorts, using the Infinium II HumanHap550K Genotyping BeadChip version 3 (Illumina Inc., San Diego, CA, USA). Genotyping procedures were followed according to the manufacturer's protocols. Microarray genotyping procedures in the Rotterdam Study have been previously described [46].*i*

Supplementary references

1. Stein,E.A. Extending therapy options in treating lipid disorders: a clinical review of cerivastatin, a novel HMG-CoA reductase inhibitor. *Drugs* **56 Suppl 1**, 25-31 (1998).

2. Thompson,J.F. *et al.* Comprehensive whole-genome and candidate gene analysis for response to statin therapy in the Treating to New Targets (TNT) cohort. *Circ. Cardiovasc. Genet.* **2**, 173-181 (2009).

3. Chasman,D.I. *et al.* Genetic Determinants of Statin Induced LDL-C Reduction: The JUPITER Trial. *Circ Cardiovasc Genet.* **5**, 257-264 (2012).

4. Donnelly,L.A. *et al.* A paucimorphic variant in the HMG-CoA reductase gene is associated with lipid-lowering response to statin treatment in diabetes: a GoDARTS study. *Pharmacogenet. Genomics* **18**, 1021-1026 (2008).

5. Akao,H. *et al.* KIF6, LPA, TAS2R50, and VAMP8 genetic variation, low density lipoprotein cholesterol lowering response to pravastatin, and heart disease risk reduction in the elderly. *Atherosclerosis* **220**, 456-462 (2012).

6. Hopewell,J.C. *et al.* Impact of common genetic variation on response to simvastatin therapy among 18 705 participants in the Heart Protection Study. *Eur. Heart J* **34**, 982-992 (2013).

7. Barber,M.J. *et al.* Genome-wide association of lipid-lowering response to statins in combined study populations. *PLoS. One.* **5**, e9763 (2010).

8. Colhoun,H.M. *et al.* Design of the Collaborative AtoRvastatin Diabetes Study (CARDS) in patients with type 2 diabetes. *Diabet. Med.* **19**, 201-211 (2002).

9. Colhoun,H.M. *et al.* Primary prevention of cardiovascular disease with atorvastatin in type 2 diabetes in the Collaborative Atorvastatin Diabetes Study (CARDS): multicentre randomised placebo-controlled trial. *Lancet* **364**, 685-696 (2004).

10. Friedewald,W.T., Levy,R.I., & Fredrickson,D.S. Estimation of the concentration of low-density lipoprotein cholesterol in plasma, without use of the preparative ultracentrifuge. *Clin. Chem.* **18**, 499-502 (1972).

11. Deshmukh,H.A. *et al.* Genome-wide association study of genetic determinants of LDL-c response to atorvastatin therapy: importance of Lp(a). *J Lipid Res.* **53**, 1000-1011 (2012).

12. Simon,J.A. *et al.* Phenotypic predictors of response to simvastatin therapy among African-Americans and Caucasians: the Cholesterol and Pharmacogenetics (CAP) Study. *Am. J Cardiol.* **97**, 843-850 (2006).

13. Albert,M.A., Staggers,J., Chew,P., & Ridker,P.M. The pravastatin inflammation CRP evaluation (PRINCE): rationale and design. *Am. Heart J* **141**, 893-898 (2001).

14. Shepherd,J. *et al.* The design of a prospective study of Pravastatin in the Elderly at Risk (PROSPER). PROSPER Study Group. PROspective Study of Pravastatin in the Elderly at Risk. *Am. J. Cardiol.* **84**, 1192-1197 (1999).

15. Shepherd,J. *et al.* Pravastatin in elderly individuals at risk of vascular disease (PROSPER): a randomised controlled trial. *Lancet* **360**, 1623-1630 (2002).

16. Trompet,S. *et al.* Replication of LDL GWAs hits in PROSPER/PHASE as validation for future (pharmaco)genetic analyses. *BMC. Med. Genet.* **12**, 131 (2011).

17. LaRosa,J.C. *et al.* Intensive lipid lowering with atorvastatin in patients with stable coronary disease. *N Engl J Med.* **352**, 1425-1435 (2005).

18. Harris,T.B. *et al.* Age, Gene/Environment Susceptibility-Reykjavik Study: multidisciplinary applied phenomics. *Am. J Epidemiol.* **165**, 1076-1087 (2007).

19. The Atherosclerosis Risk in Communities (ARIC) Study: design and objectives. The ARIC investigators. *Am. J Epidemiol.* **129**, 687-702 (1989).

20. Sharrett,A.R. *et al.* Associations of lipoprotein cholesterols, apolipoproteins A-I and B, and triglycerides with carotid atherosclerosis and coronary heart disease. The Atherosclerosis Risk in Communities (ARIC) Study. *Arterioscler. Thromb.* **14**, 1098-1104 (1994).

21. Warnick,G.R., Benderson,J., & Albers,J.J. Dextran sulfate-Mg2+ precipitation procedure for quantitation of high-density-lipoprotein cholesterol. *Clin. Chem.* **28**, 1379-1388 (1982).

22. Roden,D.M. *et al.* Development of a large-scale de-identified DNA biobank to enable personalized medicine. *Clin. Pharmacol. Ther.* **84**, 362-369 (2008).

23. Ritchie,M.D. *et al.* Robust replication of genotype-phenotype associations across multiple diseases in an electronic medical record. *Am. J Hum. Genet.* **86**, 560-572 (2010).

24. Xu,H. *et al.* MedEx: a medication information extraction system for clinical narratives. *J Am. Med. Inform. Assoc.* **17**, 19-24 (2010).

25. Fried,L.P. *et al.* The Cardiovascular Health Study: design and rationale. *Ann. Epidemiol.* **1**, 263-276 (1991).

26. Ettinger,W.H. *et al.* Lipoprotein lipids in older people. Results from the Cardiovascular Health Study. The CHS Collaborative Research Group. *Circulation* **86**, 858-869 (1992).

27. DAWBER,T.R., KANNEL,W.B., & LYELL,L.P. An approach to longitudinal studies in a community: the Framingham Study. *Ann. N Y. Acad. Sci.* **107**, 539-556 (1963).

28. KANNEL,W.B., Feinleib,M., McNamara,P.M., Garrison,R.J., & Castelli,W.P. An investigation of coronary heart disease in families. The Framingham offspring study. *Am. J Epidemiol.* **110**, 281-290 (1979).

29. Splansky,G.L. *et al.* The Third Generation Cohort of the National Heart, Lung, and Blood Institute's Framingham Heart Study: design, recruitment, and initial examination. *Am. J Epidemiol.* **165**, 1328-1335 (2007).

30. Price,A.L. *et al.* Principal components analysis corrects for stratification in genome-wide association studies. *Nat. Genet.* **38**, 904-909 (2006).

31. Doney,A.S., Lee,S., Leese,G.P., Morris,A.D., & Palmer,C.N. Increased cardiovascular morbidity and mortality in type 2 diabetes is associated with the glutathione S transferase theta-null genotype: a Go-DARTS study. *Circulation* **111**, 2927-2934 (2005).

32. Doney,A.S., Fischer,B., Leese,G., Morris,A.D., & Palmer,C.N. Cardiovascular risk in type 2 diabetes is associated with variation at the PPARG locus: a Go-DARTS study. *Arterioscler. Thromb. Vasc. Biol.* **24**, 2403-2407 (2004).

33. Donnelly,L.A. *et al.* Robust association of the LPA locus with low-density lipoprotein cholesterol lowering response to statin treatment in a meta-analysis of 30 467 individuals from both randomized control trials and observational studies and association with coronary artery disease outcome during statin treatment. *Pharmacogenet. Genomics* **23**, 518-525 (2013).

34. Bellenguez,C., Strange,A., Freeman,C., Donnelly,P., & Spencer,C.C. A robust clustering algorithm for identifying problematic samples in genome-wide association studies. *Bioinformatics.* **28**, 134-135 (2012).

35. Psaty,B.M. *et al.* The risk of myocardial infarction associated with the combined use of estrogens and progestins in postmenopausal women. *Arch. Intern. Med.* **154**, 1333-1339 (1994).

36. Psaty,B.M. *et al.* The risk of myocardial infarction associated with antihypertensive drug therapies. *JAMA* **274**, 620-625 (1995).

37. Klungel,O.H. *et al.* Antihypertensive drug therapies and the risk of ischemic stroke. *Arch. Intern. Med.* **161**, 37-43 (2001).

38. Bild,D.E. *et al.* Multi-ethnic study of atherosclerosis: objectives and design. *Am. J Epidemiol.* **156**, 871-881 (2002).

39. MRC/BHF Heart Protection Study of cholesterol lowering with simvastatin in 20,536 high-risk individuals: a randomised placebo-controlled trial. *Lancet* **360**, 7-22 (2002).

40. Ridker,P.M. *et al.* Rosuvastatin to prevent vascular events in men and women with elevated C-reactive protein. *N Engl J Med.* **359**, 2195-2207 (2008).

41. Purcell,S. *et al.* PLINK: a tool set for whole-genome association and population-based linkage analyses. *Am. J. Hum. Genet.* **81**, 559-575 (2007).

42. Li,Y., Willer,C.J., Ding,J., Scheet,P., & Abecasis,G.R. MaCH: using sequence and genotype data to estimate haplotypes and unobserved genotypes. *Genet. Epidemiol.* **34**, 816-834 (2010).

43. Hofman,A., Grobbee,D.E., de Jong,P.T., & van den Ouweland,F.A. Determinants of disease and disability in the elderly: the Rotterdam Elderly Study. *Eur. J Epidemiol.* **7**, 403-422 (1991).

44. Hofman,A. *et al.* The Rotterdam Study: 2014 objectives and design update. *Eur. J Epidemiol.* **28**, 889-926 (2013).

45. Miller,S.A., Dykes,D.D., & Polesky,H.F. A simple salting out procedure for extracting DNA from human nucleated cells. *Nucleic Acids Res.* **16**, 1215 (1988).

46. Richards,J.B. *et al.* Bone mineral density, osteoporosis, and osteoporotic fractures: a genome-wide association study. *Lancet* **371**, 1505-1512 (2008).
